# An integrative approach sheds new light onto the systematics and ecology of the widespread ciliate genus *Coleps* (Ciliophora, Prostomatea)

**DOI:** 10.1038/s41598-021-84265-y

**Published:** 2021-03-15

**Authors:** Thomas Pröschold, Daniel Rieser, Tatyana Darienko, Laura Nachbaur, Barbara Kammerlander, Kuimei Qian, Gianna Pitsch, Estelle Patricia Bruni, Zhishuai Qu, Dominik Forster, Cecilia Rad-Menendez, Thomas Posch, Thorsten Stoeck, Bettina Sonntag

**Affiliations:** 1 Research Department for Limnology, Mondsee, University of Innsbruck, Mondsee, Austria; 2 Experimental Phycology and Culture Collection of Algae, University of Göttingen, Göttingen, Germany; 3 College of Environmental Engineering, Xuzhou Institute of Technology, Xuzhou, People’s Republic of China; 4 Limnological Station, Department of Plant and Microbial Biology, University of Zürich, Kilchberg, Switzerland; 5 Laboratory of Soil Biodiversity, University of Neuchâtel, Neuchâtel, Switzerland; 6 Department of Ecology, Technical University of Kaiserslautern, Kaiserslautern, Germany; 7 Culture Collection of Algae and Protozoa, Scottish Association for Marine Science, Oban, Scotland

**Keywords:** Ecology, Evolution, Zoology, Limnology

## Abstract

Species of the genus *Coleps* are one of the most common planktonic ciliates in lake ecosystems. The study aimed to identify the phenotypic plasticity and genetic variability of different *Coleps* isolates from various water bodies and from culture collections. We used an integrative approach to study the strains by (i) cultivation in a suitable culture medium, (ii) screening of the morphological variability including the presence/absence of algal endosymbionts of living cells by light microscopy, (iii) sequencing of the SSU and ITS rDNA including secondary structures, (iv) assessment of their seasonal and spatial occurrence in two lakes over a one-year cycle both from morphospecies counts and high-throughput sequencing (HTS), and, (v) proof of the co-occurrence of *Coleps* and their endosymbiotic algae from HTS-based network analyses in the two lakes. The *Coleps* strains showed a high phenotypic plasticity and low genetic variability. The algal endosymbiont in all studied strains was *Micractinium conductrix* and the mutualistic relationship turned out as facultative. *Coleps* is common in both lakes over the whole year in different depths and HTS has revealed that only one genotype respectively one species, *C. viridis*, was present in both lakes despite the different lifestyles (mixotrophic with green algal endosymbionts or heterotrophic without algae). Our results suggest a future revision of the species concept of the genus *Coleps*.

## Introduction

**Figure 1:**
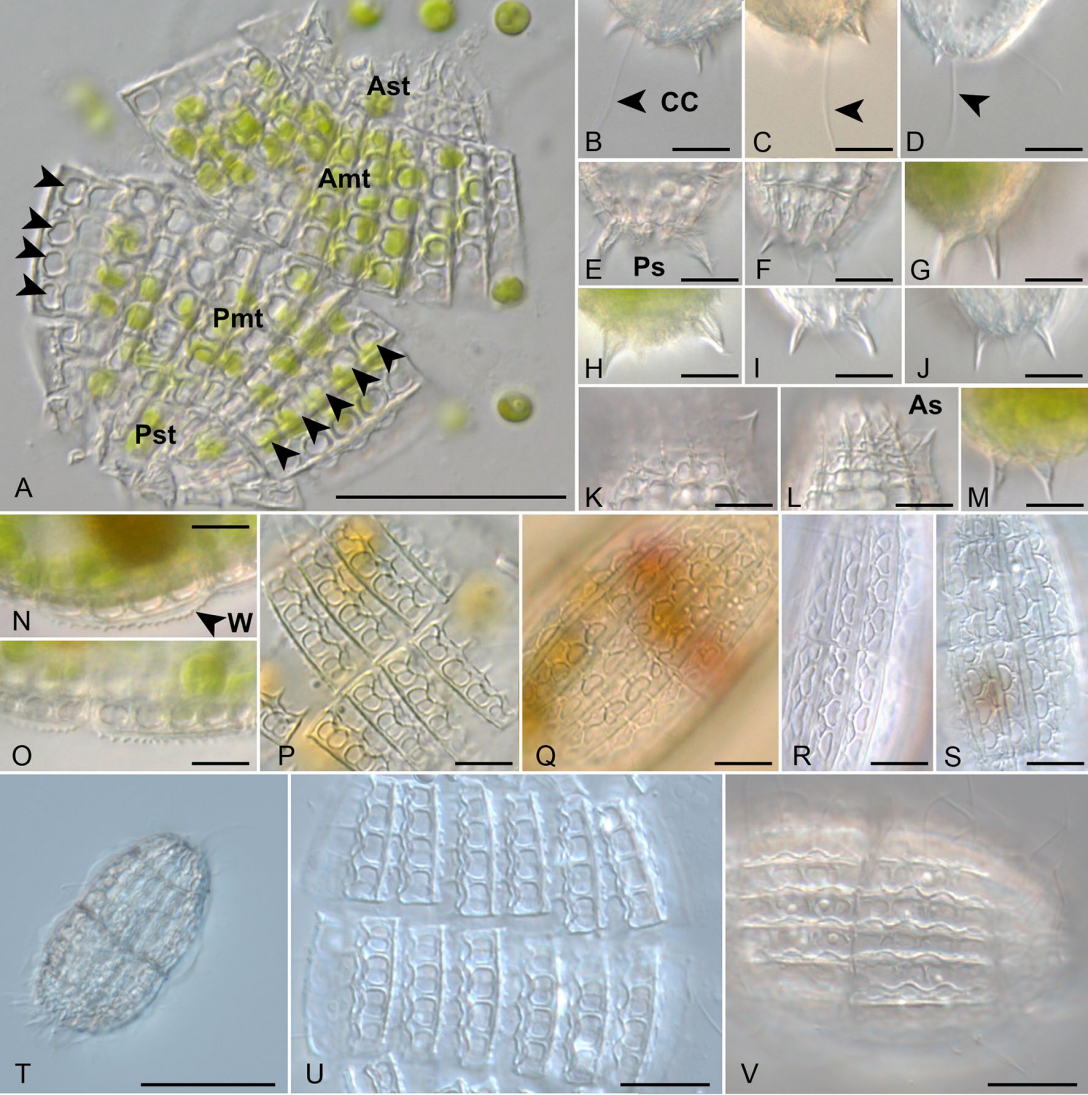
Morphology and phenotypic plasticity of *Coleps viridis* (**A–P**, **T–V**) and *Nolandia nolandi* (**Q–S**) from life. (**A**) Overall plate structure in a heavily squeezed specimen. Note the simultaneous occurrence of four and five windows in the posterior main tier (arrows), (**B–D**) Posterior portion with dorsal spines and one caudal cilium, (**E**–**J**, **M**) Posterior portion with spines, (**K**–**L**) Anterior protion with spines, (**N–O**) Lateral Wing, (**P**) Pretzel-shaped plate windows of the *hirtus*-type, (**Q–S**) Reniform plate windows of the *nolandia*-type, (**T–V**) Spine-less specimens; scale bar = 20 m (**A**), 5 μm (**B**–**S**), 30 μm (**T**), 10 μm (**U**,** V**). **Amt** anterior main tiers, **As** anterior spine, **Ast** anterior secondary tiers, **CC** caudal cilium, **Pmt** posterior main tiers, **Ps** posterior spine, **Pst** posterior secondary tiers, **W** lateral wing.

Ciliates of the genus *Coleps* Nitzsch, 1827 are widely distributed in diverse marine, brackish and freshwater habitats^1–11^. Currently, more than 20 morphological species originally assigned to *Coleps*, have been described since the first report of *C. hirtus*, the type species^12^. Later, most of them have been transferred to new genera (see details in Table S1). Colepids are morphologically relatively easily recognized due to their armored calcium carbonate plates that are characteristic for their overall appearance^1,13^ (Fig. 1). Diagnostic features for the differentiation of colepid species, which were used for descriptions of genera, are the number of the armor tiers, the structure of the tier plates, the presence/absence of armor spines, and the number of adoral organelles^12^. These morphological traits were also used for the subdivision of colepids into several genera (for detailed summary and morphological definition of the genera; see^12,14,15^). As a result of the subdivision into genera (Table S1), only five described morphospecies remained in the genus *Coleps*: *C. amphacanthus* Ehrenberg, 1833^16–20^, *C. elongatus* Ehrenberg, 1831^1,21^, *C. hirtus* Nitzsch, 1827^1,22^, *C. viridis* Ehrenberg, 1831^1^, and *C. spetai* Foissner, 1984^12^.

In freshwater habitats such as lakes and ponds, four colepid species can be frequently found even within the same water body^1–4,23–27^, i.e., *C. hirtus hirtus*, *C. hirtus viridis*, *C. spetai* and *Nolandia nolandi* Kahl, 1930 (= formerly *C. nolandi*). The morphology of these species is very similar except for the structure of the tier plates, i.e., the pretzel-shaped *hirtus*-type in the three former and the reniform *nolandi*-type in the latter species^12,16^ (Fig. 1P–S). *Coleps hirtus hirtus* and *Nolandia nolandi* are considered to be heterotrophic omnivores, whereas *C. hirtus viridis* and *C. spetai* are known to be mixotrophic because they bear endosymbiotic green algae (for an ecological summary on the four species, see Foissner et al.^1^). The latter two are so far separated by wing-like protrusions that occur as conspicuous structures in *C. spetai* only (Fig. 1N–O), whereas both *C. hirtus* subspecies can be distinguished by the grass-green appearance in *C. hirtus viridis* caused by the possession of endosymbionts^22^. Moreover, the presence of armor spines has as well been considered as a genus-specific feature in both *Coleps* and *Nolandia*^12^.

Phylogenetic analyses of the Prostomatea using SSU rDNA sequences demonstrated that the colepid taxa represented a monophyletic lineage although the generic concept within this group is questionable^7,9,28^. Even the species belonging to *Coleps* in the revision of Foissner et al.^12^ are not members of the same clade. For example, *C.* *amphacanthus* is closely related to the genus *Levicoleps*^12^, whereas *C. hirtus* and *C. spetai* are sisters of *Nolandia nolandi*^9^.

To discover the cryptic diversity within colepid species, Barth et al.^29^ studied the genetic variability of *C. hirtus*, *C. spetai* and *Nolandia nolandi* by comparing their mitochondrial apocytochrome *b* gene. The authors analyzed 111 isolates and revealed a high genetic intraspecific diversity within *C. hirtus* *hirtus* (9 haplotypes) and *C. spetai* (11 haplotypes) with a clear separation into hetero- and mixotrophic morphotypes and a sister to *Nolandia nolandi* (3 haplotypes). Cryptic diversity has also been reported for other ciliates (peritrichs^30,31^; tintinnids^32^; heterotrichs^33^).

However, the phenotypic morphological plasticity of the colepid ciliates as well as the stability of the endosymbiosis with their green algal partners (facultative or obligate) was not investigated so far. Very little is known about the identity of green algal endosymbionts in ciliates or even protists and for convenience, they were usually designated as *zoochlorellae* (see Foissner et al.^1^, for a compilation of algal-bearing planktonic ciliates). Pröschold et al.^34^ investigated one algal strain, which was isolated from *C. hirtus* and its SSU/ITS rDNA sequence was identical with the green alga *Chlorella vulgari*s, that is frequently found free-living as well as in symbiosis with other ciliates such as *Paramecium bursaria*. Interestingly, the mutualistic relationship among *P. bursaria* and its algal symbiont is flexible indicating that the ciliate can establish a symbiosis also with ‘foreign’ green algae derived from other ciliates^35^. This flexibility has been also described for an unidentified species of the marine colepid genus *Tiarina*, which bears several cryptic algal species of the genus *Zooxanthella* (previously known as *Symbiodinium* clade A) as endosymbionts^36^.

During a one-year sampling campaign, *Coleps* spp. were regularly found in the plankton of Lake Mondsee (Austria) and Lake Zurich (Switzerland). According to morphological discrepancies especially in the collected *Coleps* specimens compared to those of described species, we performed an integrative approach to gain a deeper insight into the ciliates as well as their endosymbionts and, moreover, the stability of the symbiosis. Throughout different seasons and from different depths, we isolated several *Coleps* strains with and without green algal endosymbionts and compared them to cultivated strains previously collected from other sites. We established clonal cultures of both the ciliates and the green algae (in the case of algal-bearing strains) and investigated (i) their morphology and (ii) their molecular sequences (from single cells as well as from HTS), (iii) the facultative or obligate nature of the association among the partners, (iv) their ecological preferences (co-occurrence), and, finally, (v) their seasonal and spatial distribution in the two lakes.

## Results

### Systematics and molecular phylogeny of *Coleps*

#### Morphology and phenotypic plasticity

Among the clone cultures, we found a broad spectrum of morphological variations (Table 1 + S2). Out of 19 clones comprising several *Coleps* strains and one clone of *Nolandia nolandi*, only two could be assigned at the morphospecies level following to the identification key of Foissner et al.^1^. All others showed more than 20% variation among the investigated individual clones according to their morphometric features and made a final identification questionable (Table 1). The features which fit to the descriptions of *C. hirtus* and *C. spetai*, were marked in blue and green in Table 1, respectively (constant features and differing characters are highlighted in white and in orange, respectively). The phenotypic plasticity was high among the strains, which can be seen in pie charts in Fig. 3 by the color-coding. In detail, the number of plate rings in the anterior and posterior primary and secondary plates was largely constant, the number of plate windows in the anterior and posterior secondary plates varied even within one individual, the shape of the plate windows corresponded to the *hirtus*-type and the *nolandia*-type, respectively, and, the number and presence/absence of anterior and posterior armour spines differed or they were absent at all (Fig. 1). The same was true for *N. nolandi*, variations in the number of plate windows and the presence/absence of anterior/posterior armour spines did not correspond to the description.

**Table 1:**
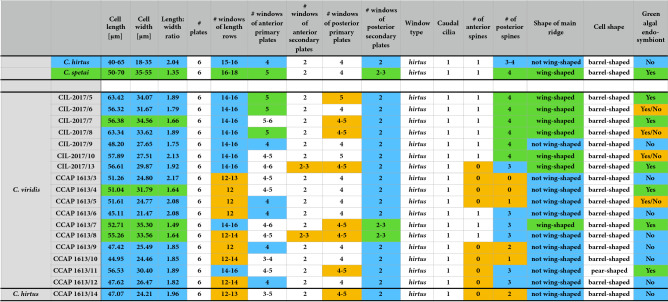
Morphological features of the investigated strains compared to those of *Coleps hirtus* (color-coded in blue) and *C. spetai* (colour-coded in green) according to Foissner^22^. Constant and divergent features are marked in white and orange, respectively.

#### SSU phylogeny of the Colepidae (Prostomatea)

**Figure 2:**
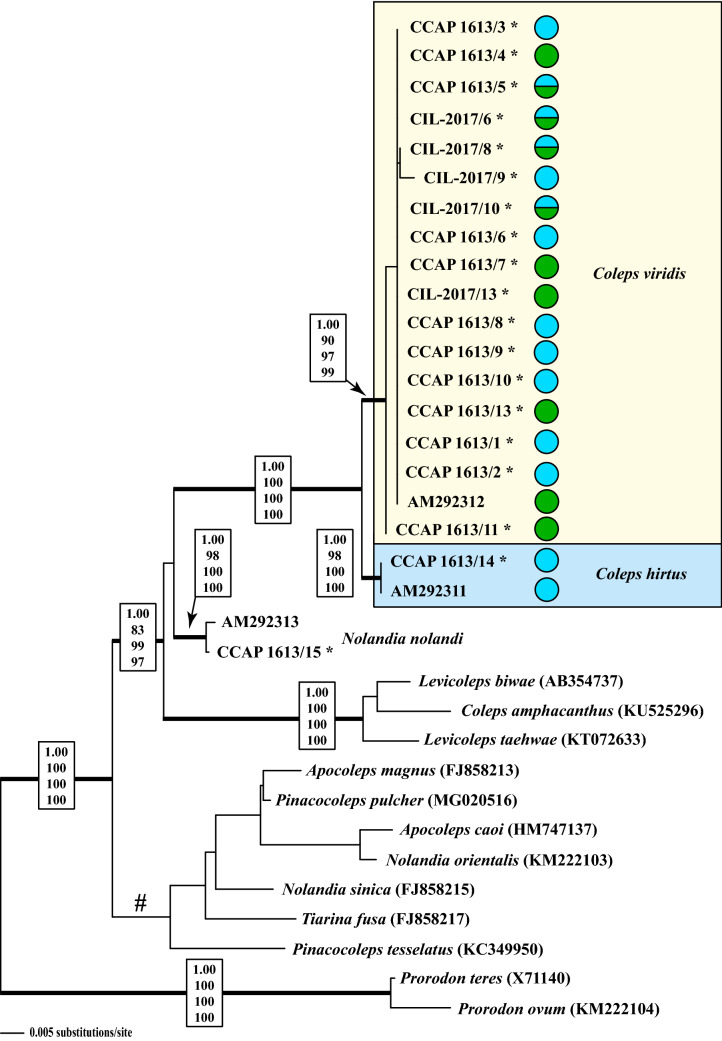
Molecular phylogeny of the Colepidae (Prostomatea) based on SSU sequence comparisons. The phylogenetic trees shown were inferred using the maximum likelihood method based on the data sets (1750 aligned positions of 34 taxa) using PAUP 4.0a166. For the analyses, the best model was calculated by Modeltest 3.7. The setting of the best model was given as follows: GTR + I + G (base frequencies: A 0.2796, C 0.1881, G 0.2526, T 0.2797; rate matrix A–C 1.0338, A–G 2.5616, A–U 1.2578, C–G 0.4974, C–U 4.5509, G–U 1.0000) with the proportion of invariable sites (I = 0.6535) and gamma shape parameter (G = 0.6457). The branches in bold are highly supported in all analyses (Bayesian values > 0.95 calculated with MrBayes, 5 million generations; bootstrap values > 70%, calculated with PAUP, 1000 replicates using maximum likelihood, neighbour-joining, and maximum parsimony). The branch marked with a hashtag was not supported in our analyses. The new sequences of this study are marked with an asterisk. The mixotrophic (with green algal endosymbionts) and heterotrophic (without algal endosymbionts) strains of *Coleps* are highlighted with green and blue circles, respectively.

The SSU and ITS rDNA of the nuclear ribosomal operon were sequenced to infer the genetic variability of the investigated strains. The SSU rDNA sequences were aligned according to the secondary structure (exemplarily presented for the strain CCAP 1613/7; Fig. S1) and incorporated into a dataset, which included all morphologically described representatives of the Colepidae (Prostomatea; Fig. 2). Our clonal strains (except for strain CCAP 1613/15 = *Nolandia nolandi*) could be assigned to two genetic groups, i.e., group 1 and group 2 (Fig. 2). The strain CCAP 1613/15 is almost identical (99.8%) to the SSU rDNA sequence of *N. nolandi* obtained by Barth et al.^29^. Group 1 includes strains with and without green algal endosymbionts (indicated by green and blue circles in Fig. 2, respectively). Only strain CCAP 1613/14 is identical with the SSU rDNA sequence of *C. hirtus hirtus* (Genbank accession number AM292311^29^) and represents group 2. Our phylogenetic analyses revealed that strains that were morphologically identified as *C. hirtus* and *C. spetai* could be found in both genetic groups. The only other already sequenced *Coleps* species, i.e., *C. amphacanthus* was not closely related to the others and genetically belonged to the genus *Levicoleps*. In addition, the other colepid genera, i.e., *Nolandia*, *Pinacocoleps* and *Levicoleps* that were represented by more than one species appeared to be also not monophyletic in our analyses and were intertwined with the genus *Tiarina*. Our findings are highly supported in all bootstrap and Bayesian analyses. Only one clade marked with a hashtag in Fig. 2 remained unresolved.

**Figure 3:**
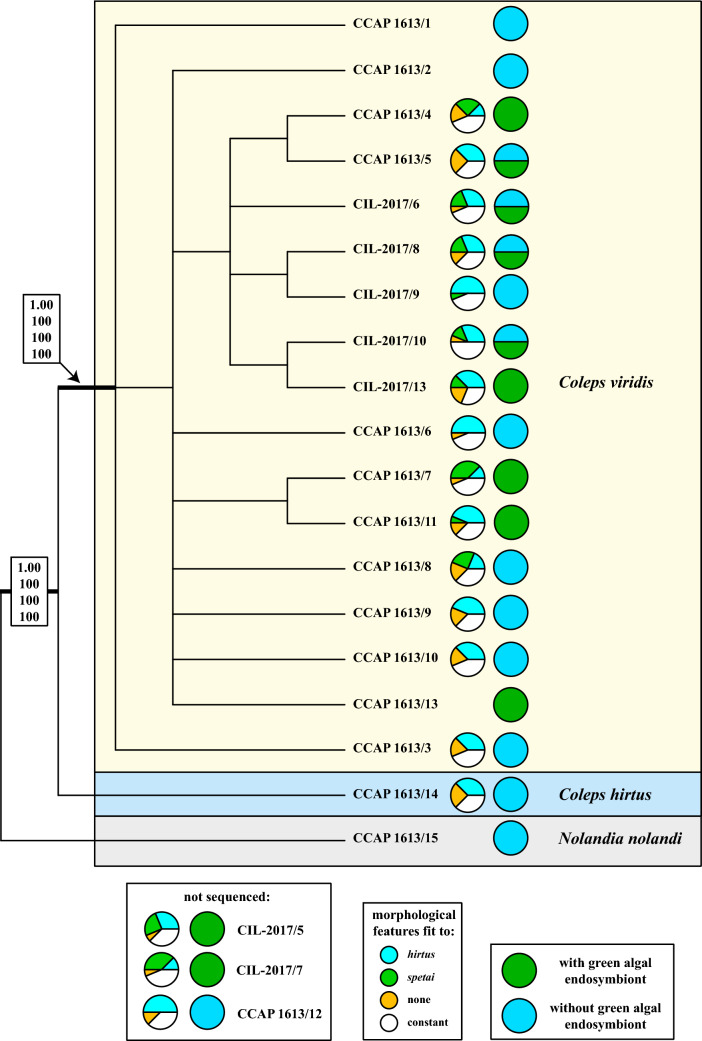
Molecular phylogeny of *Coleps* based on ITS sequence comparisons. The phylogenetic trees shown were inferred using the maximum likelihood method based on the data sets (538 aligned positions of 19 taxa) using PAUP 4.0a166. For the analyses, the best model was calculated by Modeltest 3.7. The setting of the best model was given as follows: HKY + I (base frequencies: A 0.3525, C 0.2245, G 0.1809, T 0.2421; Ti/Tv ration = 0.8758) with the proportion of invariable sites (I = 0.6535). The branches in bold are highly supported in all analyses (Bayesian values > 0.95 calculated with MrBayes, 5 million generations; bootstrap values > 70%, calculated with PAUP, 1000 replicates using maximum likelihood, neighbour-joining, and maximum parsimony). The pie charts after each strain number indicate the number of morphological features (see details in Table 1), which fitted to the characters of *C. hirtus* (blue) or *C. spetai* (green). The number of characters in white and in orange did not fit to both species. The mixotrophic (with green algal endosymbionts) and heterotrophic (without algal endosymbionts) strains of *Coleps* are highlighted with green and blue circles, respectively.

#### ITS phylogeny and ITS-2/CBC approach

**Figure 4:**
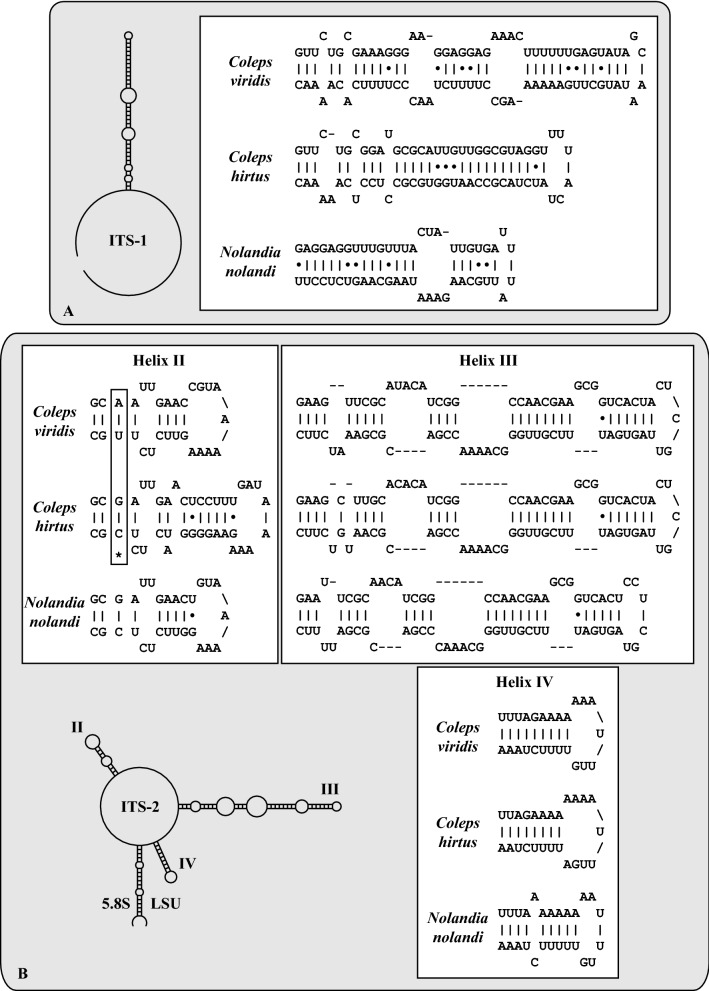
Comparisons of the ITS-1 (**A**) and ITS-2 (**B**) secondary structures among the species of *Coleps* and *Nolandia*. The line structures were drawn with PseudoViewer3^41^. The structures of the helices were calculated with mfold^42^.

**Figure 5:**
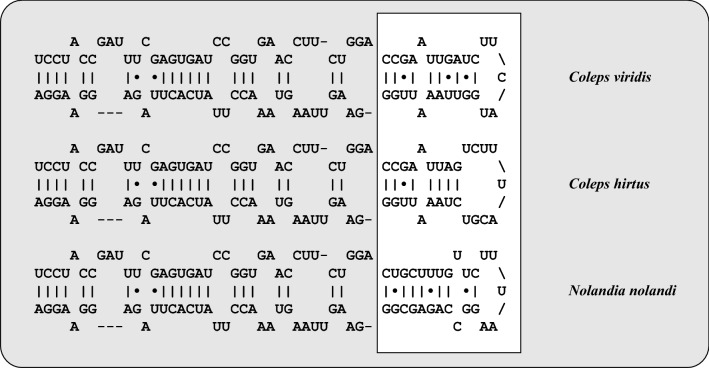
Comparisons of the V9 secondary structures among the species of *Coleps* and *Nolandia*. The structures were calculated with mfold^42^.

To decide if the two genetic groups 1 and 2 represented species, the ITS regions of the nuclear ribosomal operon were analyzed. First, the ITS-1 and ITS-2 were aligned according to their secondary structures and a phylogenetic tree was calculated using the methods described below. Second, the ITS-2 secondary structures were compared using the ITS-2/CBC approach to detect compensatory base changes (CBCs) between the groups 1 and 2. The phylogenetic analyses (Fig. 3) supported the subdivision into the two groups. The ITS-1 and ITS-2 of the investigated *Coleps* strains had a length of ~ 165 bp and ~ 210 bp, respectively, and revealed differences to the typical secondary structures presented by Coleman & Mai^37^ for green algae and plants, but similarity to the structure of *Tetrahymena tropicalis*^38^. The ITS-1 of *Coleps* formed only one helix and the ITS-2 missed the helix I but the helices II–IV were present (Fig. 4). The comparison of ITS-1 and ITS-2 secondary structures revealed that the two groups did not only differ in their structures, but also a CBC in helix II of the ITS-2 (marked with an asterisk in Fig. 4) could be observed, suggesting that both groups represented two separate species (Figs. 3 and 4). In an additional step, we tested if the two putative species could also be separated by HTS of the V9 SSU rDNA region, which is commonly used in metabarcoding studies^39,40^. Therefore, we checked the secondary structure of the investigated groups to detect their usage as marker for HTS. In Fig. 5, we demonstrated that each V9 region of the used sequences had a diagnostic part in its structure (highlighted in white).

### Systematics and molecular phylogeny of the green algal endosymbionts

#### Morphology and phenotypic plasticity of the green algal endosymbiont

**Figure 6:**
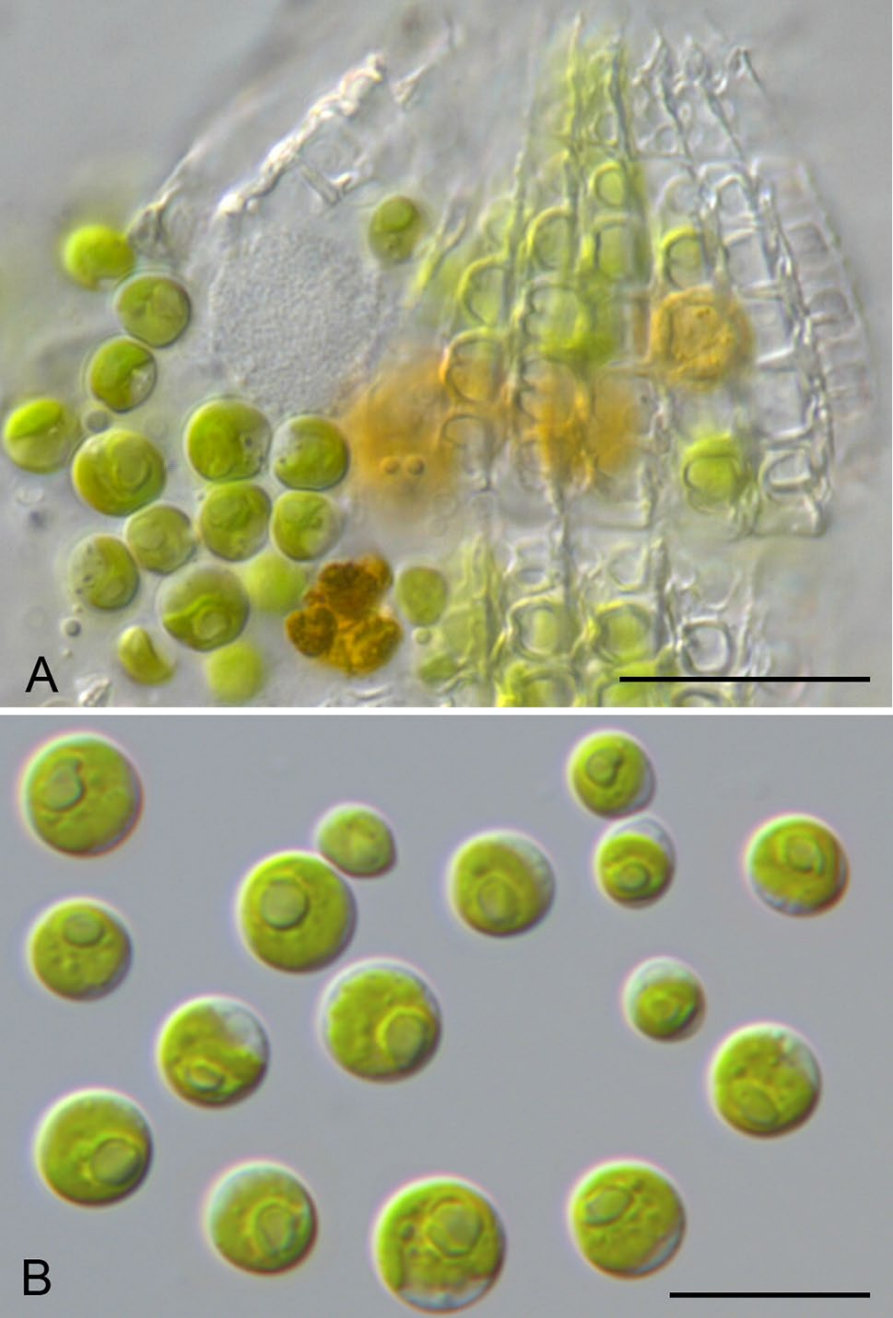
Morphology and phenotypic plasticity of *Micractinium conductrix*. (**A**) from a squeezed cell of *Coleps viridis*, strain CIL-2017/13, (**B**) isolated strain CCAP 248/20; scale bar = 10 μm.

Six *Coleps* strains belonging to group 1 had green algal endosymbionts with a *Chlorella*-like morphology (Figs. 2, 6). These unicellular green algae were characterized by a spherical to slightly ellipsoid cell shape possessing cup-shaped chloroplasts containing a single pyrenoid surrounded by two or four starch grains. The cells were 7–12 μm in diameter and reproduced asexually by 2–8 autospores. The isolated endosymbionts of three *Coleps* strains (CCAP 1613/7, CIL-2017/13 and CCAP 1613/11) did not vary in their morphological features and resembled the appearance of *Micractinium* (Fig. 6). The morphology of the isolated endosymbionts was identical to those described for *M. conductrix* in Pröschold et al.^34^.

#### SSU and ITS phylogeny of the Chlorellaceae

**Figure 7:**
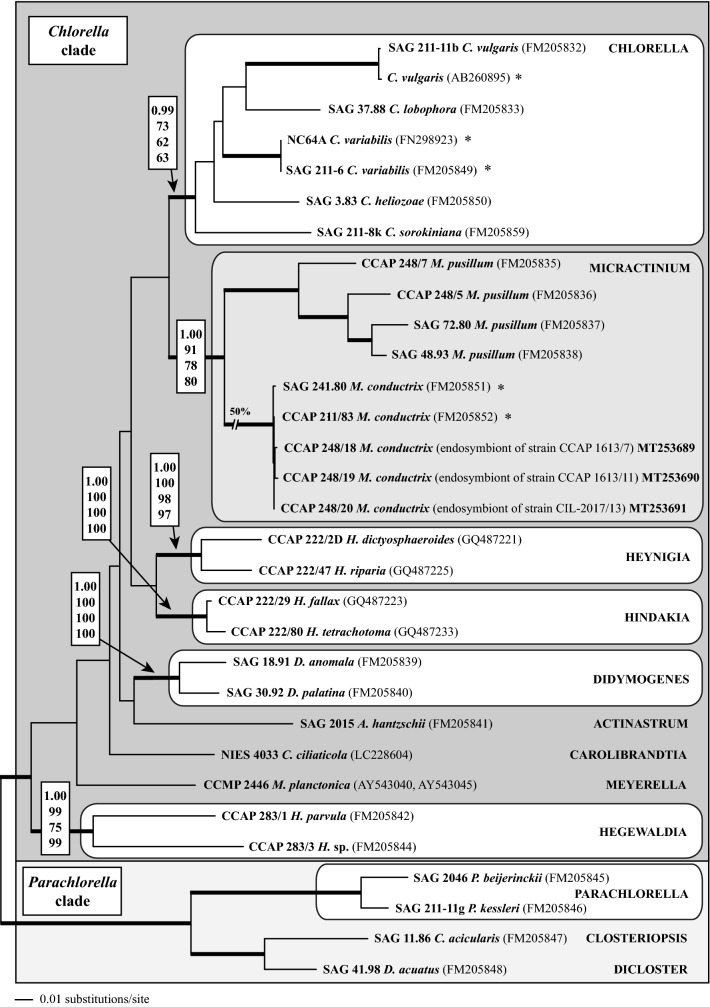
Molecular phylogeny of the Chlorellaceae based on SSU and ITS sequence comparisons. The phylogenetic trees shown were inferred using the maximum likelihood method based on the data sets (2604 aligned positions of 31 taxa) using PAUP 4.0a166. For the analyses, the best model was calculated by Modeltest 3.7. The setting of the best model was given as follows: GTR+I+G (base frequencies: A 0.2160, C 0.2732, G 0.2728, T 0.2380; rate matrix A–C 0.8499, A–G 1.2840, A–U 1.1061, C–G 0.6217, C–U 3.1850, G–U 1.0000) with the proportion of invariable sites (I = 0.6996) and gamma shape parameter (G = 0.5575). The branches in bold are highly supported in all analyses (Bayesian values > 0.95 calculated with MrBayes, 5 million generations; bootstrap values > 70%, calculated with PAUP, 1000 replicates using maximum likelihood, neighbour-joining, and maximum parsimony). The endosymbionts that were previously detected in *Paramecium bursaria* are marked with an asterisk.

The SSU and ITS rDNA sequences of the endosymbiotic algal strains were aligned according to their secondary structures and incorporated into a dataset containing representatives of the Chlorellaceae (Trebouxiophyceae). The sequences of all three strains (CCAP 248/18, CCAP 248/19, and CCAP 248/20) were almost identical (99.7%) to the strains SAG 241.80 and CCAP 211/83 (marked with an asterisk in Fig. 7) that belong to *Micractinium conductrix*, a species exclusively known as endosymbiont of *Paramecium bursaria*^34^ so far. All SSU and ITS rDNA sequences of *M. conductrix* had an intron at position 651 and varied in only seven base positions from each other (data not shown).

### Seasonal and spatial distribution of *Coleps* in two lakes

**Figure 8:**
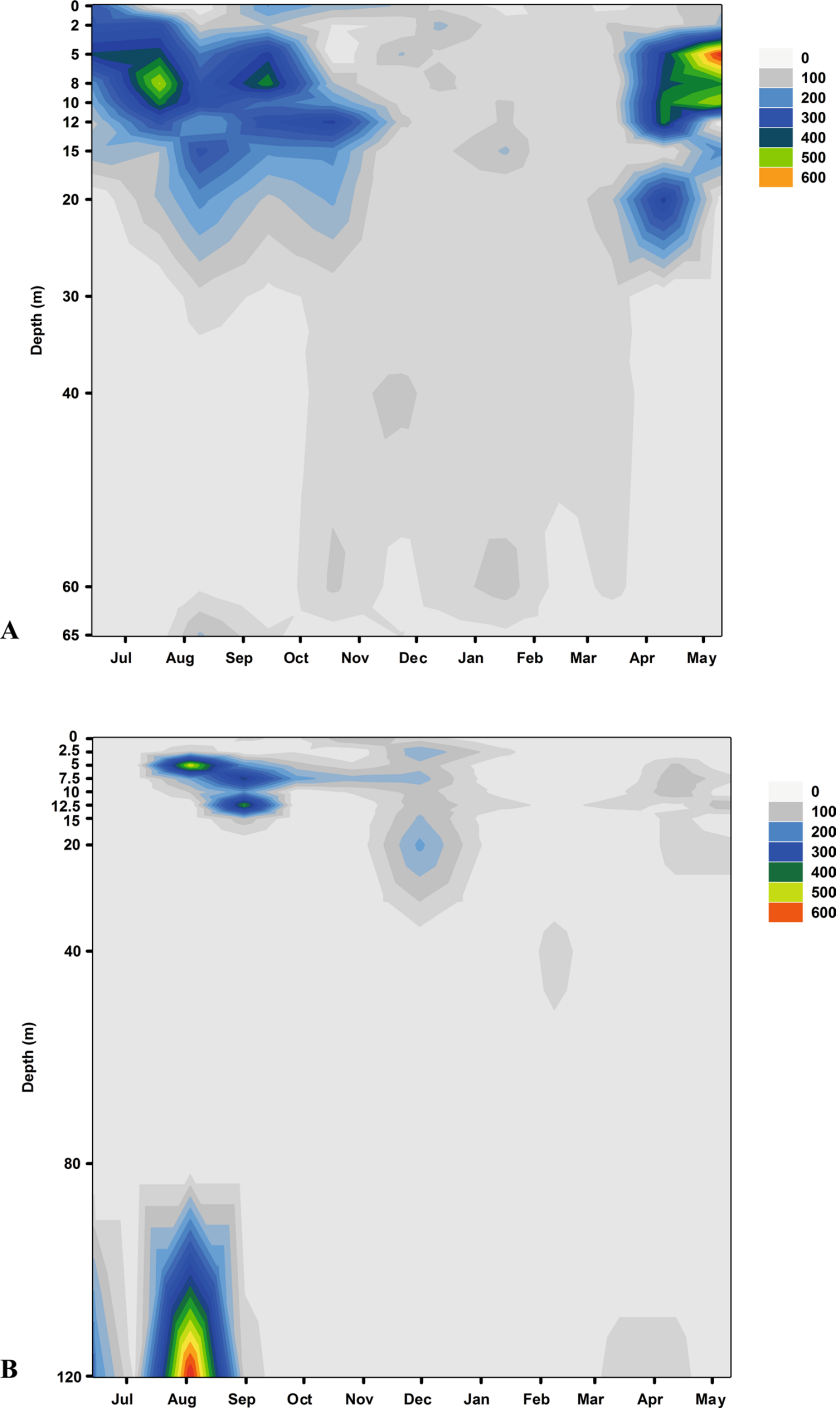
Abundance of *Coleps* in Lake Mondsee, Austria (**A**) and Lake Zurich, Switzerland (**B**). The numbers of individuals per litre were colour-coded.

*Coleps* morphotypes were found throughout the whole sampling period in Lake Mondsee in higher average numbers than in Lake Zurich (103 vs. 39 cells/L; Figs. 8, S2). Cell numbers peaked during spring and summer in Lake Mondsee, while in Lake Zurich the maximum abundance was observed once in August (Figs. 8 + S2). Algal-bearing *Coleps* morphotypes were mainly detected in the upper meters in both lakes (>80% of total *Coleps* abundance) whereas the heterotrophic ones were only found in the deepest zones (Fig. S2). *Coleps* morphotypes without algal endosymbionts were frequently found in Lake Zurich during summer but rarely in Lake Mondsee (20% vs. 1% of total abundance; Fig. S2). In contrast to Lake Zurich, a clear seasonal distribution pattern appeared among depths in Lake Mondsee. The mixotrophic *Coleps* morphotypes were more abundant during spring and summer at 5 m depth, in contrast to the deeper water layer during the cold season (Fig. S2).

The abundance patterns obtained from morphotype-observations of *Coleps* in both lakes were compared to HTS read counts of *Coleps* V9 sequences. In deeper layers, *Coleps* reads were one order of magnitude higher in Lake Mondsee than in Lake Zurich. In 5 m depth, *Coleps* reads were almost two orders of magnitude higher in Lake Mondsee than in Lake Zurich. From spring till the end of autumn, the abundance of *Coleps* reads notably increased in the 5 m depth of Lake Mondsee but decreased during winter. No seasonal succession pattern could be observed for *Coleps* reads in the deeper layers of Lake Mondsee. By contrast, seasonal *Coleps* read abundances were very similar in both layers of Lake Zurich, with a pronounced simultaneous peak in summer and another small peak in spring. Similar to Lake Mondsee, *Coleps* read abundances were lowest during winter in Lake Zurich.

### Co-occurrence networks

**Figure 9:**
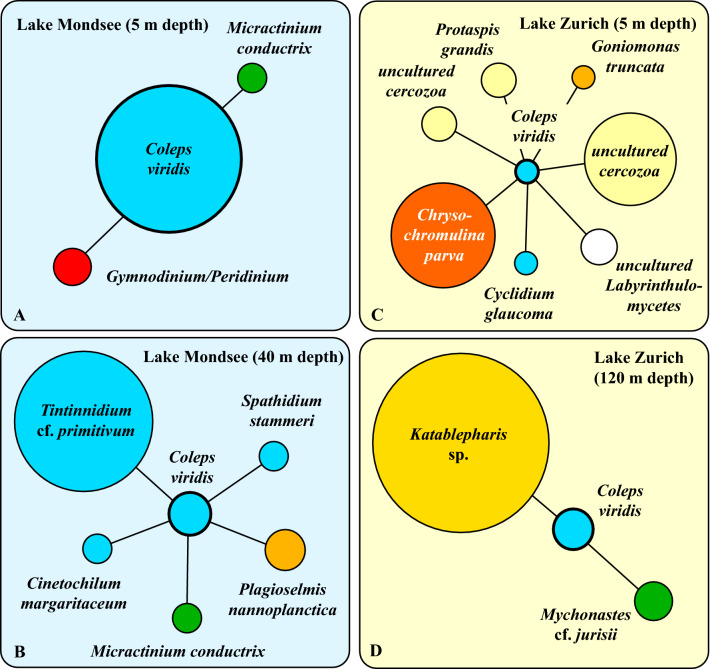
Co-occurence network of *Coleps viridis* in both lakes at two depths (**A**, **B** Lake Mondsee, Austria at 5 m and 40 m depths, respectively; **C**, **D** Lake Zurich, Switzerland at 5 m and 120 m depths, respectively) based on HTS approach using the V9 region of the SSU.

In the co-occurrence networks based on HTS-data of the two depths in both lakes, we only found *Coleps* group 1 (i.e., *C. viridis*) and not group 2 (i.e.,*C. hirtus*; see below). Moreover, we identified a significant relationship between the symbiotic green alga *M. conductrix* and *C. viridis* only in Lake Mondsee (Fig. 9). Apart from *M. conductrix*, significant correlations in Lake Mondsee were discovered with one cryptophyte (*Plagioselmis nannoplanctica*), one dinoflagellate (*Gymnodinium*/*Peridinium*) and several ciliates (*Cinetochilum margaritaceum*, *Spathidium stammeri*, *Tintinnidium* cf. *primitivum*). In Lake Zurich, heterotrophic and autotrophic flagellates (*Chrysochromulina parva*, *Goniomonas truncata*, *Kathablepharis* sp.), cercozoa (some uncultured ones and *Protaspis grandis*), stramenopiles (uncultured Labyrinthulomycetes) and one ciliate (*Cyclidium glaucoma*) were detected as significant co-occurring protists along with *C. viridis* (Fig. 9).

## Discussion

### Morphology and phenotypic plasticity

The morphological features of the investigated colepid strains differed from those described for *C. hirtus*, *C. spetai* and even for *N. nolandi*^1^ (Fig. 1). Characteristics that matched the descriptions were the ciliate cell length and width, the barrel-shaped cell (except for strain CIL-2017/7, which was pear-shaped and strain CCAP 1613/15 that had a cylindrical shape), a number of six armor tiers, the structure of the armor tiers (*hirtus*-type or *nolandia*-type, respectively), and one caudal cilium (Table S2). Variations (CV > 20%) were found (i) in the number of plate windows in the posterior/anterior main plates even within individual cells, and (ii) in the presence/absence of anterior and posterior spines (Tables 1 + S2, Fig. 1). This phenotypic plasticity of the ciliate could also be observed in freshly collected *Coleps* specimens and was therefore not an artifact resulting from cultivation conditions (Fig. 1A). Wickham and Gugenberger^43^ hypothesized that the formation of the spines was a response to grazing pressure on *C. hirtus*; however, this could not be confirmed by respective experiments. Nevertheless, spineless specimens of *C. hirtus* have obviously been found before^44–47^. Luckily, we were able to investigate two strains (CCAP 1613/1 and CCAP 1613/2) that had been kept in the CCAP culture collection since the 1950ies and the 1960ies and which did not bear any spines or symbionts and could be clearly assigned to *C. hirtus* (Fig. 1T–V). These observations suggest that without predation pressure, colepid ciliates probably do not need to synthesize spines avoiding ingestion by a predator.

The presence/absence of green algal endosymbionts, one of the diagnostic features for the discrimination among *C. hirtus* subspecies and *C. spetai*, was also not a stable feature (Table S2). Under culture conditions, some strains lost their endosymbionts completely, other strains consisted of symbiotic and aposymbiotic individuals, and some strains showed only symbiont-bearing individuals (e.g., CCAP 1613/5 and CIL-2017/6). This indicates that the symbiosis is facultative and might be probably influenced by cultivation or environmental conditions (presumably, though not tested, food availability). Consequently, the morphological separation of *C. hirtus* into the two subspecies may no longer be valid. We clearly demonstrated that the morphological features used for species descriptions can vary and have severe consequences for colepid species identification. Moreover, even the strains belonging to the groups 1 and 2 discovered by the phylogenetic analyses (Fig. 2) cannot discriminate morphotypes because they can neither be assigned to a certain cell morphology nor to the possession of algal endosymbionts. This questions the traditional morphology-based taxonomy. The separation of *Coleps hirtus hirtus*, *C. hirtus viridis* and *C. spetai*, which Foissner et al.^1^ differentiated by the presence of zoochlorellae in the latter two species and the number of windows in the armor plates, could not be supported by our analyses. *C. hirtus viridis* was originally described by Ehrenberg^48,49^ as *C. viridis* and later transferred as synonym of *C. hirtus* by Kahl^50^ based on almost identical morphological features. However, Foissner^22^ described *C. spetai* for the green *Coleps* because of the morphological discrepancies to the Ehrenberg’s *C. viridis* (presence of only 11 windows per plate row and smaller cell size in *C. viridis*; see Table 1 for comparison). Our study has clearly demonstrated that most of the morphological features are variable and the limits for species separation were too narrow. Therefore, we propose the re-establishment of *C. viridis* for group 1 and *C. hirtus* for group 2, both with emended descriptions as follows. Considering our findings, the morphological descriptions of *C. spetai*, *C. hirtus viridis* and *C. hirtus hirtus* cannot be applied for (sub-) species separation any more. Consequently, we deal with a cryptic species complex, i.e., two genetically different groups that are fused in a highly variable morphotype including features of all three (sub-) species. To solve this taxonomic problem, two possible scenarios can be proposed: (1) We merge the three morphotypes under *C. hirtus*, the type species of *Coleps*. As a consequence, two new species needed to be proposed for both groups 1 and 2, which could be done following the suggestion of Sonneborn^51^ for the *P. aurelia*-complex. However, Sonneborn based his new descriptions on results of mating experiments, which are not applicable for *Coleps* here because conjugations have not been reported and the conditions for the induction of sexual reproduction are unknown. (2) To avoid confusion by introducing new species names, we propose keeping the already existing names, i.e., *C. viridis* for group 1 and *C. hirtus* for group 2 including the synonyms (see below).

Clonal cultures of both genetically varying *Coleps* groups have been deposited in the CCAP culture collection. Future studies may therefore be able to investigate, for example, sibling among strains or predator-prey experiments revealing spine- or wing-formation.

***Coleps viridis*** Ehrenberg 1831 (printed 1832), *Abh. Königl. Akad. Wiss. Berlin* **1832**: 101.

**Synonym:** *Coleps spetai* Foissner 1984, *Stapfia* **12**: 21-22, Fig. 7, **SP**: 1984/10 and 1984/11 (lectotype designated here deposited in **LI**, see Aescht 2008: *Denisia* **23**: 179), *Coleps hirtus sensu* Kahl 1930, Tierwelt Deutschlands 18: 134.

**Diagnosis**: Differed from other colepid ciliates by their SSU and ITS rDNA sequences (MT253680).

**Lectotype** (designated here): Fig. II, Tab. XXXIII, 3 in Ehrenberg 1838, *Infusionsthierchen als vollkommene Organismen*, p. 314.

**Improved Description** (specifications in brackets apply to our reference strain CCAP 1613/7): *Coleps* with conspicuous armor composed of six tiers with plate windows of the *hirtus*-type. With or without green algal endosymbionts. Cell size 44–63 × 21–35 μm (52–54 × 35–36 μm). Total number of windows in length rows 12–16 (14–16), number of windows of anterior primary plates 3–6 (4–6), number of windows of anterior secondary plates 2–3 (2), number of windows of posterior primary plates 4–5 (4–5), number of windows of posterior secondary plates 2–3 (2–3). One caudal cilium (1). With 0-2 anterior (0–1) and 0–5 posterior (1–4) spines, respectively.

**Reference material** (designated here for HTS approaches): The reference strain CCAP 1613/7 permanently cryopreserved at CCAP in a metabolically inactive stage.

**Locality of reference strain**: Plankton of Lake Mondsee, Upper Austria, Austria (47° 50′ N, 13° 23′ E).

***Coleps hirtus*** (O.F. Müller) Nitzsch ex Ersch & Gruber 1827, *Allgemeine Encyclopädie der Wissenschaften und Künste* **16**: 69, **NT** (proposed by Foissner 1984, *Stapfia* **12**: 22, fig. 8): 1984/12 and 1984/13 (**LI**, in Aescht 2008: *Denisia* **23**: 159).

**Protonym:** *Cercaria hirta* O.F. Müller 1786, *Animalcula Infusoria*: 128, tab. XIX, fig. 17, 18 (lectotype designated here).

**Diagnosis**: Differed from other colepid ciliates by their SSU and ITS rDNA sequences (MT253687).

**Improved Description**: *Coleps* with spiny armor composed of six tiers with plates of the *hirtus*-type. Without green algal endosymbionts. Cell size 42–52 × 23–28 μm. Total number of windows in length rows 12-13, number of windows of anterior primary plates 3-5, number of windows of anterior secondary plates 2, number of windows of posterior primary plates 4-5, number of windows of posterior secondary plates 2. One caudal cilium. Without anterior and 1-4 posterior spines, respectively.

**Reference strain** (designated here for HTS approaches): The strain CCAP 1613/14 permanently cryopreserved at CCAP in a metabolically inactive stage.

**Locality of reference strain**: Plankton of Lake Piburg, Tyrol, Austria (47° 11′ N, 10° 53′ E).

### Molecular phylogeny of the Colepidae (Prostomatea)

The colepids belonging to the Prostomatea form a monophyletic lineage in the phylogenetic analyses of SSU rDNA sequences (Fig. 2). Mixotrophic as well as heterotrophic *Coleps* strains that resembled *C. hirtus* and *C. spetai* clustered in group 1 whereas group 2 included only two specimens which were identified as *C. hirtus*. These findings confirm the results of Barth *et al.*^29^ with one exception. The authors found a clear separation into mixotrophic and heterotrophic species, which were therefore assigned to a *C. spetai*-(with endosymbionts) and a *C. hirtus*-group (without endosymbionts), respectively. Despite the difficulties of identifying these species by morphology, both groups clearly differed in their SSU and ITS rDNA sequences (Fig. 3). The ITS-2/CBC approach introduced for green algae (details in Darienko et al.^52^) clearly demonstrated that both groups represented two separate ciliate species from a molecular point of view, which was also confirmed by analyses of the V9 region of the SSU, a region commonly used for metabarcoding (Figs. 4 and 5).

Our study also confirmed the findings of Chen et al.^7^, Lu et al.^9^, and Moon et al.^28^, showing that the generic concept of colepid ciliates needs to be revised. None of the genera represented by more than one species is monophyletic. For example, the three species of *Nolandia* belonged to separate lineages. *Nolandia nolandi* was a sister to our studied strains, whereas both other species were closely related to taxa of *Apocoleps*, *Pinacocoleps*, and *Tiarina* (Fig. 2). The genus *Levicoleps* and *Coleps amphacanthus* formed a monophyletic clade representing another example that the generic conception is artificial and needs to be revised. However, to provide a new generic concept of colepid ciliates, it is necessary to study more of the described species by using an integrative approach including experimental approaches on, e.g., the formation of spines. For example, we clearly demonstrated that one key feature, which is the presence/absence of anterior/posterior spines, is highly variable and can therefore not be used to separate colepid genera as indicated by Foissner et al.^12^ (Fig. 1). There is a need for more experimental studies with colepids belonging to the *Cyclidium viridis* and *C. hirtus* morphotype. Therefore, we deposited all clones used in this study in the CCAP culture collection. One option would be to incorporate all species into one genus, i.e., *Coleps* in revised form.

### Endosymbiosis in *Coleps*

Some strains of *Coleps* are known to bear green algal endosymbionts^1^. These green algae have *Chlorella*-like morphology (Fig. 6) and were identified as *Micractinium conductrix* (Fig. 7). So far, this alga was only known as endosymbiont of the ciliate *Paramecium bursaria*^34^. All green algal endosymbionts of *Coleps* harbored this *Micractinium* species. In contrast, Pröschold et al.^34^ found that one ciliate strain identified as *C. hirtus viridis* had *Chlorella vulgaris* as endosymbiont (the algae has been deposited in the Culture Collection of Algae and Protozoa under the number CCAP 211/111). Unfortunately, this ciliate strain is not available anymore^53^.

### Ecology and distribution

For limnological studies, the preservation with Bouin’s solution and QPS is an appropriate method for quantifying and identifying ciliate species in environmental samples^54^. However, the quality in characterization of ciliates at the species level is sometimes limited as, in case of *Coleps,* the characteristic armored calcium carbonate plates are dissolved by the acidified fixation solution. Therefore, in our study, we could only distinguish between algal-bearing (mixotrophic) and non-algal-bearing (heterotrophic) *Coleps*. Despite that limitation, we could clearly see that the heterotrophic ones were only found in the deepest zones of both lakes (Fig. 8A). Not surprisingly, *Coleps* is often observed in nutrient- and ion-rich and also oxygen-depleted freshwater habitats or areas, e.g., sulfurous and crater lakes^1,5,6,27,55,56^ or even in the sludge of wastewater treatment plants^57^. Mixotrophic individuals of *Coleps* were mainly found in the upper layers of both lakes, whereas in Lake Mondsee we could also detect specimens down to 40 m depth (Fig. 8). In contrast to the mero- and monomictic Lake Zurich^4,10,58,59^, Lake Mondsee is holo- and dimictic^60^. During mixis events, algal-bearing *Coleps* specimens can be transferred passively from the upper layers into the deeper zones and vice versa. Although morphotype countings and HTS analyses reads matched quite well, we found discrepancies that have already been discussed before^10,61^ (Fig. S2).

### Biogeographic aspects (haplotype network)

**Figure 10:**
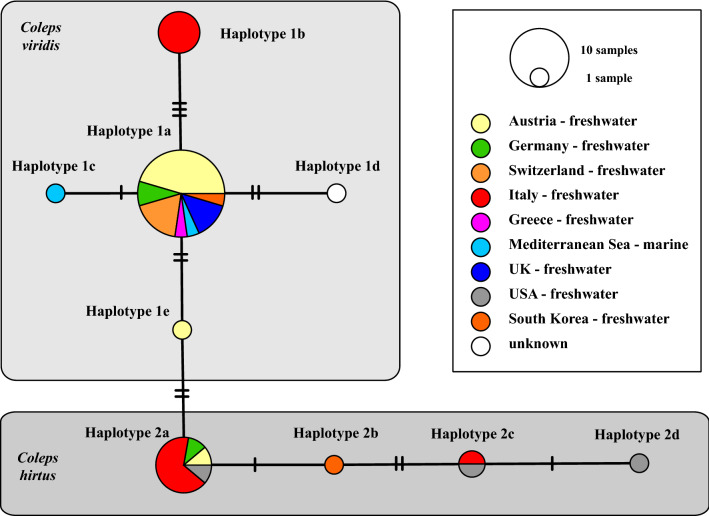
TCS haplotype network inferred from V4 sequences of *Coleps viridis* and *C. hirtus*. This network was inferred using the algorithm described by Clement et al.^64,65^. Sequence nodes corresponding to samples collected from different geographical regions and from different habitats.

Our metabarcoding approach showed that *C. viridis* was found in both lakes as a common ciliate (Fig. S2). In contrast, *C. hirtus* could not be detected during the sampling period. To obtain more information about the distribution of both species, we used the BLASTn search algorithm^62^ (100 coverage, >97% identity) for the V4 and the V9 regions of the SSU and the ITS-2 sequences. No records using the V9 and the ITS-2 approaches could be discovered in GenBank, but 25 reference sequences using the V4 (Table S3). Together with the newly sequenced strains, we therefore constructed a V4 haplotype network (Fig. 10). Both groups are obviously widely distributed and subdivided into five (group 1) and four (group 2) haplotypes, respectively. All reference sequences were collected from freshwater habitats except for two marine records^63^ (EU446361 and EU446396; Mediterranean Sea) and showed no geographical preferences.

### Co-occurrence networks

In the sub-networks of *C. viridis* in both lakes, we found several significant correlations that pointed to either potential prey items, e.g., diverse flagellated autotrophic or heterotrophic protists or co-occurring ciliates (Fig. 9). Also, the smaller ciliates such as *Cinetochilum margaritaceum* or *Cyclidium glaucoma* may as well be considered as food for the omnivorous *C. viridis* (for a compilation of the food spectrum; see Foissner *et al.*^1^). However, we identified the endosymbiont *M. conductrix* and its host *C. viridis* from both sub-networks of Lake Mondsee but not of Lake Zurich (Fig. 9). Despite this result, we want to point out that we may probably not find *M. conductrix* free-living in a water body because outside their ciliate host the algae were immediately attacked and killed by so-called *Chlorella*-viruses^66^. Therefore, the HTS-detection of *M. conductrix* was probably only together with a host ciliate. This might further explain why the green algae were detected in Lake Mondsee even in the aphotic 40 m zone where photosynthesis was impossible and individuals probably passively transferred into the deeper area by lake mixis.

### Outlook

As demonstrated in our study, the combination of traditional morphological investigations, which includes the phenotypic plasticity of the cloned strains, and modern molecular analyses using both SSU and ITS sequencing as well as HTS approaches advise a taxonomic revision of the genus *Coleps*. This comprehensive and integrative approach is also applicable for other ciliate species and genera and will provide new insights into the ecology and evolution of this important group of protists.

### Experimental procedures

#### Study sites, lake sampling and origin of the *Coleps* strains

Our main study sites were Lake Mondsee (Austria) and Lake Zurich (Switzerland), two pre-alpine oligo-mesotrophic lakes that were sampled at the deepest point of each lake (Table S4). Water samples were taken monthly from June 2016 through May 2017 over the whole water column and additionally biweekly at two main depths, i.e., 5 m in both lakes, 40 m in Lake Mondsee, and 120 m in Lake Zurich, respectively. A 5-L-Ruttner water sampler was used for Lake Zurich and a 10-L-Schindler-Patalas sampler (both from Uwitec, Austria) for Lake Mondsee. Twelve *Coleps* strains were isolated from Lake Mondsee and one from Lake Zurich. Another six clones could be obtained either from already successfully cultivated own strains, fresh isolates or from culture collections. Detailed information about sampling sites, dates and strain numbers is given in Table S2.

#### Seasonal and spatial distribution and abundance

For quantification, subsamples (200-300 mL) were preserved with Bouin’s solution (5% f.c.) containing 15 parts of picric acid, 5 parts of formaldehyde (37%) and 1 part of glacial acetic acid^54^. The samples were filtered through 0.8 μm cellulose nitrate filters (Sartorius, Germany) equipped with counting grids. The ciliates were stained following the protocol of the quantitative protargol staining (QPS) method after Skibbe^54^ with slight modifications after Pfister et al.^67^. The permanent slides were analyzed by light microscopy up to 1600x magnification with a Zeiss Axio Imager.M1 and an Olympus BX51 microscope. For identification of *Coleps* and *Nolandia* cells, the identification key of Foissner et al.^1^ was used. Microphotographs were taken with a ProgRes C14 plus camera using the ProgRes Capture Pro imaging system (version 2.9.0.1, Jenoptik, Jena, Germany).

#### Cloning, identification and cultivation of ciliates and endosymbionts

Single cells of *Coleps* were isolated and washed using the Pasteur pipette method^68^. The isolated strains were cultivated in 400 μl modified Woods Hole medium^69^ (MWC; modified) and Volvic mineral water in a mixture of 5:1 and with the addition of 10 μl of an algal culture (*Cryptomonas* sp., strain SAG 26.80) as food in microtiter plates. These clonal cultures were transferred into larger volumes after successful enrichment. All cultures were maintained at 15–21 °C under a light: dark cycle of 12:12 h (photon flux rate up 50 mol m^−2^ s^−1^).

For the isolation of their green algal endosymbionts, single ciliates were washed again and transferred into fresh MWC medium. After starvation and digestion of any food, after approx. 24 hrs, cells were washed again and the ciliates transferred onto agar plates containing Basal Medium with Beef Extract (ESFl; medium 1a in Schlösser^70^). Before placement of the ciliates onto agar plates, 50 μm of an antibiotic mix (mixture of 1% penicillin G, 0.25% streptomycin, and 0.25% chloramphenicol) were added to prevent bacterial growth. The agar plates were kept under the same conditions as described. After growth (6–8 weeks), the algal colonies were transferred onto agar slopes (1.5%) containing ESFl medium and kept under the described culture conditions.

For light microscopic investigations of the algae, Olympus BX51 and BX60 microscopes (equipped with Nomarski DIC optics) were used. Microphotographs were taken with a ProgRes C14 plus camera using the ProgRes Capture Pro imaging system (version 2.9.0.1, Jenoptik, Jena, Germany).

#### PCR, sequencing and phylogenetic methods

Single-cell PCR was used to obtain the sequences of the *Coleps* strains. Before PCR amplification, single cells of *Coleps* were washed as described above. After starvation followed by additional washing steps, cells were transferred into 5 μm sterile water in PCR tubes and the prepared PCR mastermix containing the primers EAF3 and ITS055R^71^ was added. After this primary PCR amplification and subsequent PCR purification, a nested PCR was conducted using the primer combinations EAF3/N1400R and N920F/ITS055R^71^.

The sequences of the *Coleps* strains were aligned according to their secondary structures of the SSU and ITS rDNA (see detailed folding protocol described in Darienko et al.^52^) and included into two data sets: (i) 34 SSU rDNA sequences (1,750 bp) of representatives of all members of the Prostomatea and (ii) 19 ITS rDNA sequences (538 bp) of the investigated strains. Genomic DNA of the green algae was extracted using the DNeasy Plant Mini Kit (Qiagen GmbH, Hilden, Germany). The SSU and ITS rDNA were amplified using the Taq PCR Mastermix Kit (Qiagen GmbH, Hilden, Germany) with the primers EAF3 and ITS055R. The SSU and ITS rDNA sequences of the isolated green algae (aligned according to the secondary structures) were included into a data set of 31 sequences (2,604 bp) of representatives of the Chlorellaceae (Trebouxiophyceae).

GenBank accession numbers of all newly deposited sequences can be found in Table S2 and in Fig. 7, respectively. For the phylogenetic analyses, the datasets with unambiguously aligned base positions were used. To test which evolutionary model fit best for both data sets, we calculated the log-likelihood values of 56 models using Modeltest 3.7^72^ and the best models according to the Akaike criterion by Modeltest were chosen for the analyses. The settings of the best models are given in the figure legends. The following methods were used for the phylogenetic analyses: distance, maximum parsimony, maximum likelihood, and Bayesian inference. Programs used included PAUP version 4.0b164^73^, and MrBayes version 3.2.3^74^.

The secondary structures were folded using the software mfold^42^, which uses the thermodynamic model (minimal energy) for RNA folding.

#### Haplotype networks

The haplotypes of the V4 region were identified among the groups of *Coleps* (see Fig. S1). The present haplotypes and the metadata (geographical origin and habitat) of each strain belonging to the different haplotypes are given in Table S3. To establish an overview on the distribution of the *Coleps* groups, the V4 haplotypes were used for a BLASTn search^62^ (100% coverage, >97% identity). To construct the haplotype networks, we used the TCS network tool^64,65^ implemented in PopART^75^.

#### High-throughput sequencing of the V9 18S rDNA region and subsequent bioinformatic analyses

On each sampling date, water samples for a high-throughput sequencing approach (HTS) were taken in depths of 5 m and 40 m at Lake Mondsee and 5 m and 120 m depths in Lake Zurich. DNA extraction, amplification of the V9 SSU rDNA, HTS and quality filtering of the obtained raw reads was conducted as described in Pitsch et al.^10^. After quality filtering, all remaining reads were subjected to a two-level clustering strategy^76^. In the first level, replicated reads were clustered in SWARM version 2.2.2 using *d*=1^77^. In the second level, the representative sequences of all SWARM OTUs were subjected to pairwise sequence alignments in VSEARCH version 2.11.0^78^ to construct sequence similarity networks at 97% sequence similarity. The network sequence clusters (NSCs) resulting from the second level of clustering were then taxonomically assigned by running BLASTn analyses against NCBI’s GenBank flat-file release version 230.0 and the *Coleps* SSU sequences obtained from single-cell sequencing. Network sequence clusters were assigned to *Coleps*, if the closest BLAST hit of the NSC representative sequence was a *Coleps* reference sequence. Furthermore, the NSC representative sequence had to share a fragment of at least 48 consecutive nucleotides and at least 90% sequence similarity to a reference sequence in order to be assigned to *Coleps*.

#### Co-occurrence networks

With the protist community data matrix resulting from HTS, we further conducted co-occurrence network analyses to assess biotic and abiotic interactions of *Coleps*. For each lake and depth, we ran network analyses with NetworkNullHPC (https://github.com/lentendu/NetworkNullHPC) following the null model strategy developed by Connor et al.^79^. This strategy was especially designed for dealing with HTS datasets and allows for inferring statistically significant correlations between NSCs while minimizing false positive correlation signals. We screened the resulting networks for *Coleps* nodes and extracted their subnetworks including all directly neighbouring co-occurrence partners as well as all edges between *Coleps* and its neighbours and the neighbours themselves.

## Supplementary Information

Supplementary Information.
