# Supplementary material for: An integrative approach sheds new light onto the systematics and ecology of the widespread ciliate genus Coleps (Ciliophora, Prostomatea)
Source: Sci Rep. 2021 Mar 15;11:5916. doi: 10.1038/s41598-021-84265-y (PMC7960993; doi:10.1038/s41598-021-84265-y)
Supplement: Supplementary file 1 — Supplementary Information. [file 41598_2021_84265_MOESM1_ESM.pdf]

Supplemental Material to:

An integrative approach sheds new light onto the systematics and ecology of the widespread ciliate genus *Coleps* (Ciliophora, Prostomatea)

Thomas Pröschold[1], Daniel Rieser[1], Tatyana Darienko[2], Laura Nachbaur[1], Barbara Kammerlander[1], Kuimei Qian[1,3], Gianna Pitsch[4], Estelle Patricia Bruni[4,5], Zhishuai Qu[6], Dominik Forster[6], Cecilia Rad-Menendez[7], Thomas Posch[4], Thorsten Stoeck[6] & Bettina Sonntag[1]

[1] Research Department for Limnology, Mondsee, University of Innsbruck, Mondsee, Austria

[2] Experimental Phycology and Culture Collection of Algae, University of Göttingen, Germany

[3] College of Environmental Engineering, Xuzhou Institute of Technology, Xuzhou, People's Republic of China

[4] Limnological Station, Department of Plant and Microbial Biology, University of Zürich, Kilchberg, Switzerland

[5] Laboratory of Soil Biodiversity, University of Neuchâtel, Neuchâtel, Switzerland

[6] Department of Ecology, Technical University of Kaiserslautern, Kaiserslautern, Germany

[7] Culture Collection of Algae and Protozoa, Scottish Association for Marine Science, Oban, Scotland

Figure S1: SSU secondary structure of *Coleps viridis*, strain CCAP 1613/7 (MT253680). The V4 and V9 regions of the SSU are highlighted.

SSU rRNA secondary structure model
of *Coleps viridis*
(CCAP 1613/7)
Accession number: MT253680

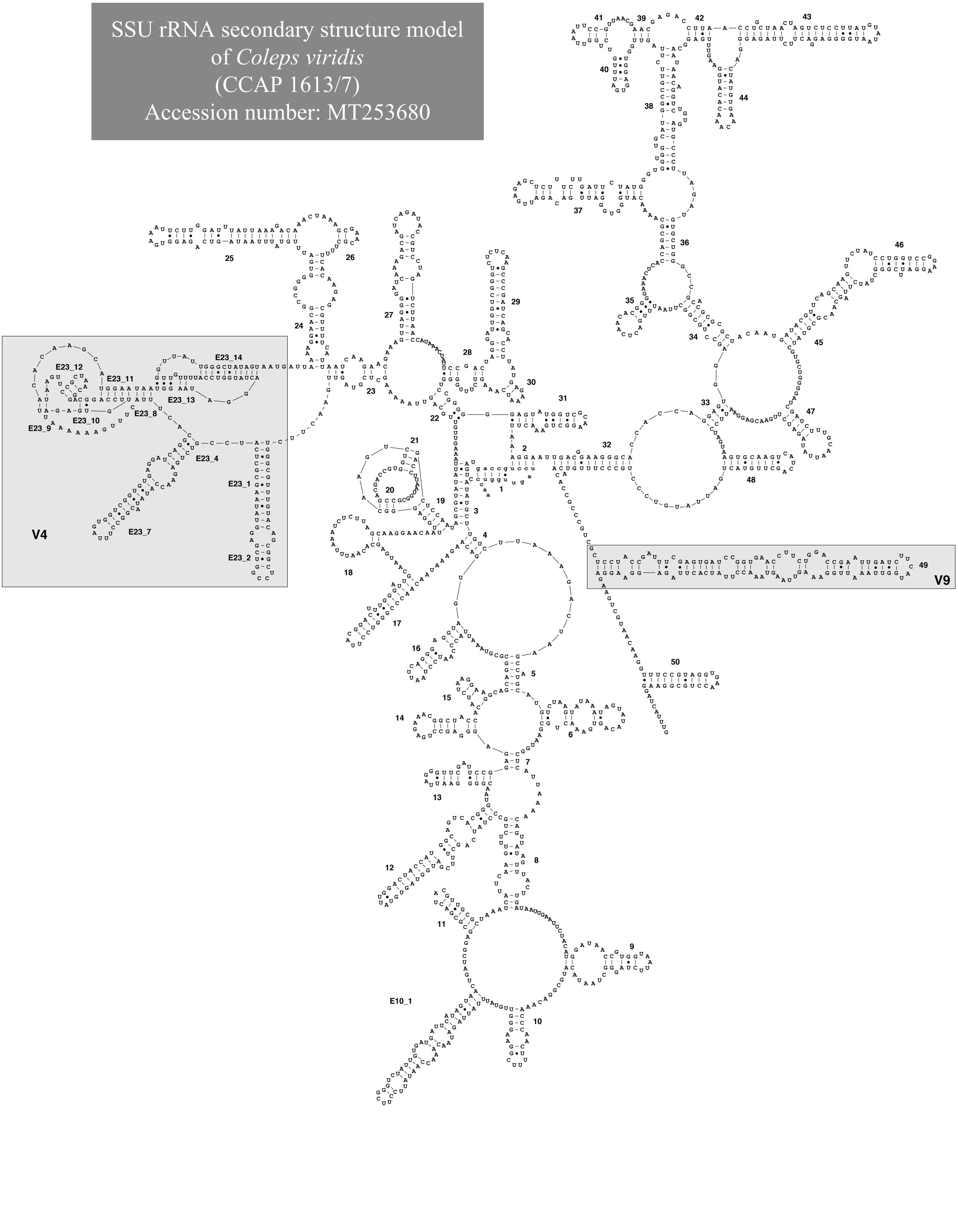

Figure S2: Comparison between abundance of *Coleps viridis* and the number of reads of this species and its endosymbiotic green algae *Micractinium conductrix* in Lake Mondsee (LM) and Lake Zurich (LZ).

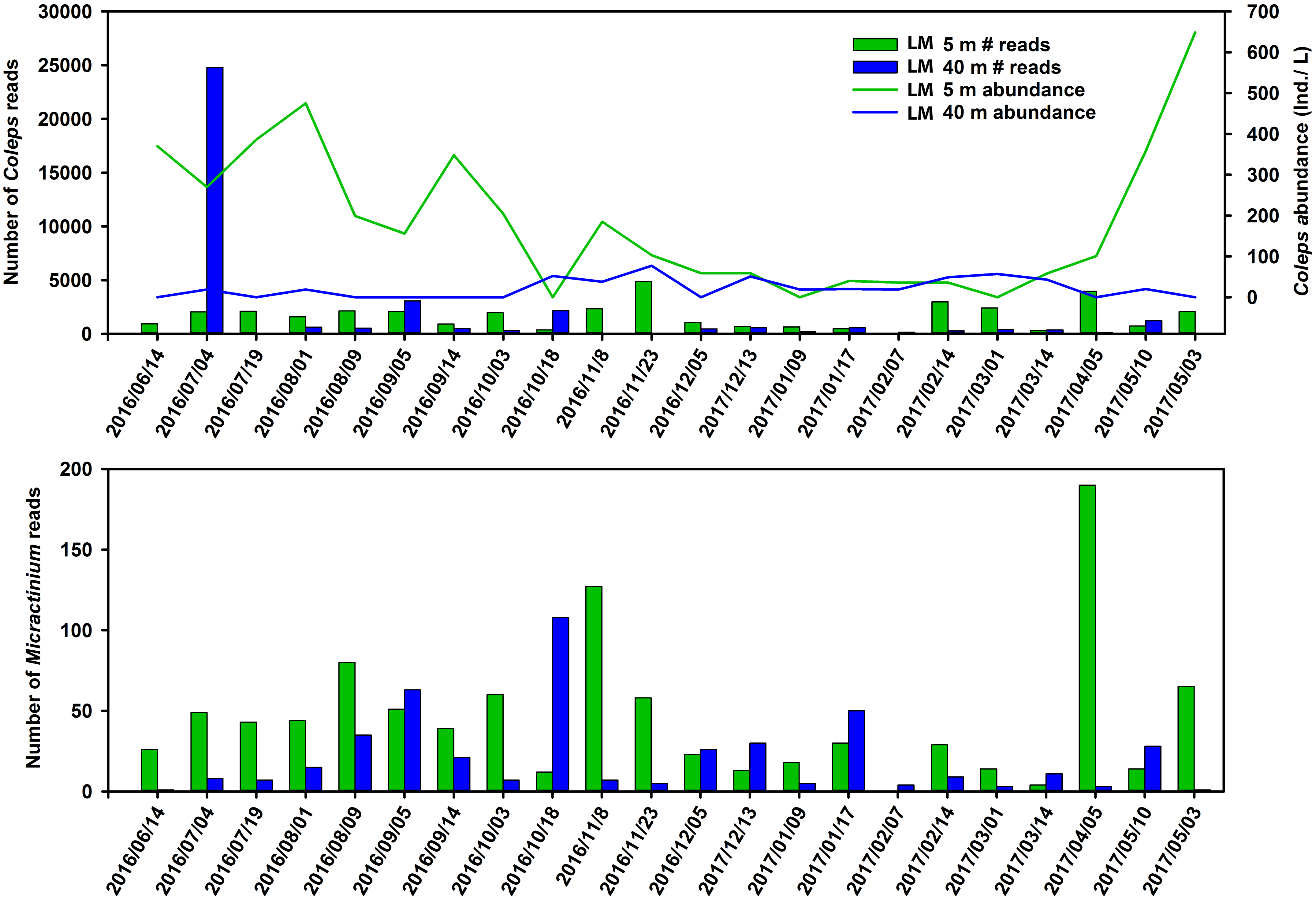

Number of Coleps reads
Coleps abundance (Ind./ L)
LM 5 m # reads
LM 40 m # reads
LM 5 m abundance
LM 40 m abundance
0
5000
10000
15000
20000
25000
30000
0
100
200
300
400
500
600
700
2016/06/14
2016/07/04
2016/07/19
2016/08/01
2016/08/09
2016/09/05
2016/09/14
2016/10/03
2016/10/18
2016/11/8
2016/11/23
2016/12/05
2017/12/13
2017/01/09
2017/01/17
2017/02/07
2017/02/14
2017/03/01
2017/03/14
2017/04/05
2017/05/10
2017/05/03
Number of Micractinium reads
0
50
100
150
200
2016/06/14
2016/07/04
2016/07/19
2016/08/01
2016/08/09
2016/09/05
2016/09/14
2016/10/03
2016/10/18
2016/11/8
2016/11/23
2016/12/05
2017/12/13
2017/01/09
2017/01/17
2017/02/07
2017/02/14
2017/03/01
2017/03/14
2017/04/05
2017/05/10
2017/05/03

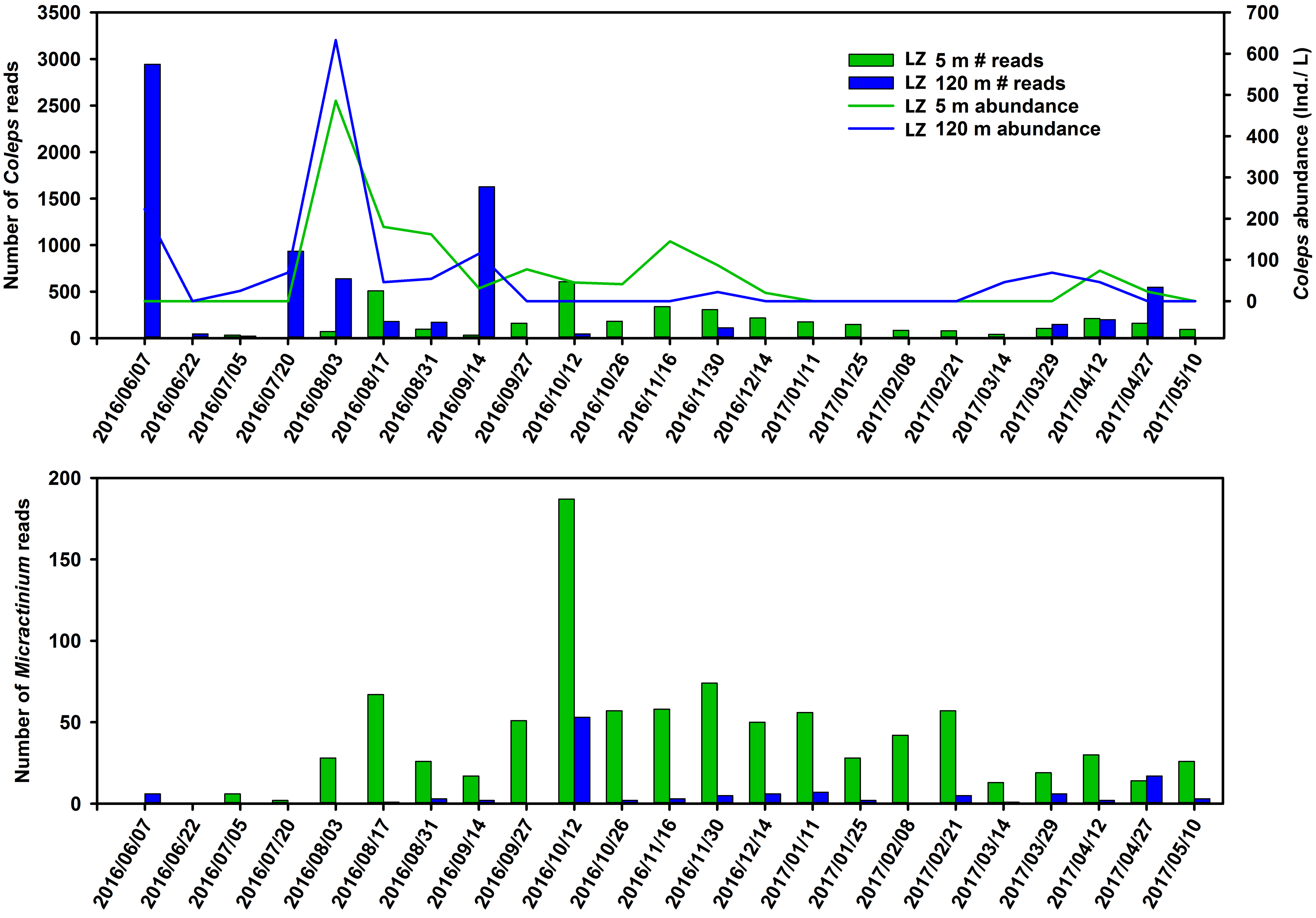

Number of Coleps reads
Coleps abundance (Ind./ L)
LZ 5 m # reads
LZ 120 m # reads
LZ 5 m abundance
LZ 120 m abundance
0
500
1000
1500
2000
2500
3000
3500
0
100
200
300
400
500
600
700
2016/06/07
2016/06/22
2016/07/05
2016/07/20
2016/08/03
2016/08/17
2016/08/31
2016/09/14
2016/09/27
2016/10/12
2016/10/26
2016/11/16
2016/11/30
2016/12/14
2017/01/11
2017/01/25
2017/02/08
2017/02/21
2017/03/14
2017/03/29
2017/04/12
2017/04/27
2017/05/10
Number of Micractinium reads
0
50
100
150
200
2016/06/07
2016/06/22
2016/07/05
2016/07/20
2016/08/03
2016/08/17
2016/08/31
2016/09/14
2016/09/27
2016/10/12
2016/10/26
2016/11/16
2016/11/30
2016/12/14
2017/01/11
2017/01/25
2017/02/08
2017/02/21
2017/03/14
2017/03/29
2017/04/12
2017/04/27
2017/05/10

Table S1: Overview over described genera belonging to the Colepidae (Prostomatea). For *Coleps* and *Tiarina* symbiotic associations with photosynthetic protists are known. All other genera contain heterotrophic congeners.

Colepid genera	Colepidae with	plate type	species #	reference
Coleps Nitzsch 1827	spiny armour composed of 6 tiers	***hirtus***	5	Foissner et al. (2008), Lu et al. (2016)
Kotinia Obolkina in Aescht 2001	spiny armour composed of 8 tiers	unspecified	3	Aescht (2001)
Levicoleps Foissner, Kusuoka & Shimano 2008	smooth armour composed of 6 tiers	***hirtus***	2	Foissner et al. (2008), Chen et al. (2016)
Macrocoleps Obolkina 1995	spiny armour composed of about 12 irregular tiers	unspecified	2	Obolkina (1995)
Nolandia Small & Lynn 1985	spiny armour composed of 6 tiers	***nolandi***	2	Small & Lynn (1985), Chen et al. (2010), Chen et al. (2012)
Pinacocoleps Diesing 1865	spiny armour composed of 6 tiers	***incurvus***	7	Diesing (1865), Chen et al. (2010), Lu et al. (2013), Moon et al. (2017)
Planicoleps Dragesco & Dragesco-Kernéis 1991	smooth armour composed of 8 tiers	unspecified	1	Dragesco & Dragesco-Kernéis (1991)
Reticoleps Foissner, Kusuoka & Shimano 2008	spiny armour composed of 6 tiers	***remanei***	1	Foissner et al. (2008)
Tiarina Bergh 1881	spiny/smooth armour composed of 6 tiers	***tiarina***	1	Bergh (1881), Chen et al. (2012)
Apocoleps Chen, Warren & Song 2009	spiny armour composed of 8 tiers	***nolandi***	2	Chen et al. (2009), Chen et al. (2012)

References:

Aescht, E. Catalogue of the generic names of ciliates (Protozoa, Ciliophora). *Denisia* **1**, 1-350 (2001).

Bergh, R. S. Bidrag til Cilioflagellaternes Naturhistorie. *Videnskabel. Meddel. fr. D. Naturhist. Forening Kjøbenhavn* **3**, 60–76 (1881).

Chen, X., Warren, A. & Song, W. Taxonomic studies on a new marine ciliate, Apocoleps magnus gen. nov., spec. nov. (Ciliophora, Colepidae), isolated from Qingdao,China. *J. Ocean Univ. China* **8**, 317-321 (2009).

Chen, X., Wang, Y., Long, H., Al-Rasheid, K. A. S., Warren, A. & Song, W. Morphological studies on two marine colepid ciliates from Qingdao, China, *Nolandia orientalis* spec. nov. and *Pinacocoleps similis* (Kahl, 1933) comb. nov. (Ciliophora, Colepidae). *Eur. J. Protistol.* **46**, 254-262 (2010).

Chen, X., Gao, S., Liu, W., Song, W., Al-Rasheid, K. A. S. & Warren, A. Taxonomic descriptions of three marine colepid ciliates, *Nolandia sinica* spec. nov., *Apocoleps caoi* spec. nov. and *Tiarina fusa* (Claparede & Lachmann, 1858) Bergh, 1881 (Ciliophora, Prorodontida). *Internat. J. Syst. Evol. Microbiol.* **62**, 735-744 (2012).

Chen, X., Sharib, S. U. A., Kim, J. H., Jang, S. W. & Shin, M. K. Morphological description and molecular phylogeny of two species of *Levicoleps* (Ciliophora, Prostomatida), *L. taehwae* nov. spec. and *L. biwae jejuensis* nov. subspec., collected in Korea. *J. Eukaryot. Microbiol.* **63**, 471-480 (2016).

Diesing, K. M. Revision der Prothelminthen. Abtheilung: Amastigen. I. Amastigen ohne Peristom. *Sber. Akad. Wiss. Wien* **52**, 505-579 (1865).

Dragesco, J. & Dragesco-Kernéis, A. Free-living ciliates from the coastal area of Lake Tanganyika (Africa). *Eur. J. Protistol.* **26**, 216-235 (1991).

Foissner, W., Kusuoka, Y. & Shimano, S. Morphology and gene sequence of *Levicoleps biwae* n. gen., n. sp. (Ciliophora, Prostomatida), a proposed endemic from the ancient Lake Biwa, Japan. *J. Eukaryot. Microbiol.* **55**, 185-200 (2008).

Lu, B., Huang, J. & Chen, X. The morphology and SSU rRNA gene sequence analysis of a poorly-known brackish water ciliate, *Pinacocoleps tesselatus* (Kahl, 1930) (Ciliophora, Colepidae) from Hangzhou Bay, China. *Zootaxa* **3637**, 123-130 (2013)

Lu, B.-R., Ma, M.-Z., Gao, F., Shi, Y.-H. & Chen, X.-R. Morphology and molecular phylogeny of two colepid species from China, *Coleps amphacanthus* Ehrenberg, 1833 and *Levicoleps biwae jejuensis. Zool. Res.* **37**, 176-185 (2016).

Moon, J. H., Kim, J. H. & Jung, J.-H. Taxonomical reinvestigation of the colepid species *Pinacocoleps pulcher* (Spiegel, 1926) Foissner *et al.*, 2008 (Ciliophora: Prorodontida: Colepidae). *Acta Protozool.* **56**, 161-169 (2017).

Obolkina, L. A. New species of the family Colepidae (Prostomatida, Ciliophora) from Lake Baikal. *Zool. Zh.* **74**, 3-19 (1995).

Small, E. B. & Lynn, D. H. Phylum Ciliophora Doflein, 1901. In: Lee, J. J., Hutner, S. H. & Bovee, E. C. (eds), *An illustrated guide to the protozoa*. Society of Protozoologists, Lawrence, KS, pp. 393-575 (1985).

Table S2: Origin and morphometric characteristics and accession numbers of the investigated *Coleps* strains. All measurements in µm. (Mean = arithmetic mean; SD = standard deviation; SE = standard error; CV = coefficient of variation in %; Min. = minimum; Max. = maximum; n = number of specimens analyzed).

Species	Strain number	Origin	Country	Morphology studied	Accession #
Coleps viridis	**CIL-2017/5**	Lake Mondsee, Upper Austria, 0-20m	Austria	yes	-
	**CIL-2017/6**	Lake Mondsee, Upper Austria, 30-63m	Austria	yes	MT253675
	**CIL-2017/7**	Lake Mondsee, Upper Austria, 30-63m	Austria	yes	-
	**CIL-2017/8**	Lake Mondsee, Upper Austria, 0-20m	Austria	yes	MT253676
	**CIL-2017/9**	Lake Mondsee, Upper Austria, 30-63m	Austria	yes	MT253677
	**CIL-2017/10**	Lake Mondsee, Upper Austria, 0-20m	Austria	yes	MT253678
	**CIL-2017/13**	Lake Mondsee, Upper Austria, 0-20m	Austria	yes	MT253681
	**CCAP 1613/1**	Garden pool, Shelford, Cambridge	England	no	MT253670
	**CCAP 1613/2**	Brick pits, Madingley, Cambridge	England	no	MT253671
	**CCAP 1613/3**	Manchester	England	yes	MT253672
	**CCAP 1613/4**	Gattiker pond, Canton Zurich	Switzerland	yes	MT253673
	**CCAP 1613/5**	Lohbach, Tyrol	Austria	yes	MT253674
	**CCAP 1613/6**	Lake Mondsee, Upper Austria, 30-63m	Austria	yes	MT253679
	**CCAP 1613/7**	Lake Mondsee, Upper Austria, 0-20m	Austria	yes	MT253680
	**CCAP 1613/8**	Lake Zurich, Canton Zurich	Switzerland	yes	MT253682
	**CCAP 1613/9**	Lake Mondsee, Upper Austria, 30-63m	Austria	yes	MT253683
	**CCAP 1613/10**	Trough at the station of National Park Heiliges Meer, Nordrhein-Westfalen	Germany	yes	MT253684
	**CCAP 1613/11**	Lake Mondsee, Upper Austria, 30-63m	Austria	yes	MT253685
	**CCAP 1613/12**	Lake Mondsee, Upper Austria, 30-63m	Austria	yes	-
Coleps hirtus	**CCAP 1613/14**	Lake Piburg, Tyrol	Austria	yes	MT253687
Nolandia nolandi	**CCAP 1613/15**	Lake Wirpitsch, Salzburg	Austria	yes	MT253688

Character	Species	Strain number	Mean	Median	SD	SE	CV	Min.	Max.	n
Cell length	*Coleps viridis*	CIL-2017/5	63.29	63.42	1.01	0.22	1.59	61.35	64.9	21
		CIL-2017/6	56.13	56.32	0.90	0.19	1.60	54.48	57.4	21
		CIL-2017/7	56.46	56.38	0.88	0.19	1.56	55.07	58.61	21
		CIL-2017/8	63.37	63.34	0.50	0.11	0.80	62.44	64.5	21
		CIL-2017/9	48.02	48.2	0.61	0.13	1.27	46.64	48.98	21
		CIL-2017/10	57.90	57.89	0.46	0.10	0.81	57.17	58.75	21
		CIL-2017/13	56.63	56.61	0.41	0.09	0.73	56.1	57.6	21
		CCAP 1613/3	51.70	51.26	1.49	0.32	2.88	49.37	55.39	21
		CCAP 1613/4	51.14	51.04	0.96	0.21	1.89	49.02	52.7	21
		CCAP 1613/5	51.34	51.61	0.98	0.21	1.91	48.8	52.65	21
		CCAP 1613/6	45.03	45.11	0.64	0.14	1.42	44.01	45.96	21
		CCAP 1613/7	52.81	52.71	0.53	0.11	1.02	51.95	53.82	21
		CCAP 1613/8	55.01	55.26	0.75	0.16	1.36	53.16	56.12	21
		CCAP 1613/9	47.43	47.42	0.58	0.12	1.23	46.15	48.48	21
		CCAP 1613/10	44.99	44.95	0.52	0.11	1.17	44.21	45.9	21
		CCAP 1613/11	56.70	56.53	0.45	0.09	0.79	56.07	57.7	21
		CCAP 1613/12	47.58	47.62	0.51	0.11	1.08	46.18	48.75	21
	Coleps hirtus	CCAP 1613/14	47.31	47.07	2.59	0.56	5.48	42.28	51.91	21
	Nolandia nolandi	CCAP 1613/15	43.57	43.6	0.63	0.13	1.46	42.12	44.83	21

Character	Species	Strain number	Mean	Median	SD	SE	CV	Min.	Max.	n
Cell width	*Coleps viridis*	CIL-2017/5	34.76	34.07	2.22	0.48	6.41	31.76	40.52	21
		CIL-2017/6	31.37	31.67	1.27	0.27	4.04	28.26	33.26	21
		CIL-2017/7	34.56	34.39	0.70	0.15	2.04	33.52	35.77	21
		CIL-2017/8	33.65	33.62	0.40	0.08	1.19	33.12	34.65	21
		CIL-2017/9	27.75	27.65	0.50	0.11	1.82	27.07	28.91	21
		CIL-2017/10	27.43	27.51	0.33	0.07	1.21	26.88	27.96	21
		CIL-2017/13	29.88	29.87	0.45	0.09	1.52	29.11	30.94	21
		CCAP 1613/3	24.54	24.8	2.06	0.45	8.42	20.61	29.55	21
		CCAP 1613/4	31.79	31.4	1.69	0.37	5.34	29.16	35.07	21
		CCAP 1613/5	24.77	24.73	0.87	0.19	3.55	23.45	26.8	21
		CCAP 1613/6	21.49	21.47	0.33	0.07	1.56	21.03	22.29	21
		CCAP 1613/7	35.33	35.3	0.20	0.04	0.58	34.92	35.71	21
		CCAP 1613/8	33.44	33.56	0.87	0.19	2.60	32.07	34.82	21
		CCAP 1613/9	25.51	25.49	0.29	0.06	1.14	25.1	26.17	21
		CCAP 1613/10	24.61	24.46	0.40	0.08	1.63	24.15	25.57	21
		CCAP 1613/11	30.31	30.4	0.43	0.09	1.43	29.18	30.9	21
		CCAP 1613/12	26.49	26.47	0.54	0.11	2.05	25.06	27.66	21
	Coleps hirtus	CCAP 1613/14	24.53	24.21	1.35	0.29	5.53	22.64	27.69	21
	Nolandia nolandi	CCAP 1613/15	21.69	22.26	1.21	0.26	5.58	19.53	23.38	21

Character	Species	Strain number	Mean	Median	SD	SE	CV	Min.	Max.	n
Length:width ratio	*Coleps viridis*	CIL-2017/5	1.82	1.85	0.10	0.02	5.73	1.58	1.93	21
		CIL-2017/6	1.79	1.76	0.08	0.01	4.81	1.68	2.02	21
		CIL-2017/7	1.63	1.64	0.03	0	2.12	1.54	1.68	21
		CIL-2017/8	1.88	1.88	0.02	0	1.42	1.81	1.91	21
		CIL-2017/9	1.73	1.72	0.04	0	2.40	1.64	1.78	21
		CIL-2017/10	2.11	2.10	0.02	0	1.23	2.07	2.15	21
		CIL-2017/13	1.89	1.89	0.03	0	1.73	1.81	1.95	21
		CCAP 1613/3	2.12	2.13	0.22	0.04	10.37	1.73	2.68	21
		CCAP 1613/4	1.61	1.62	0.07	0.01	4.77	1.43	1.73	21
		CCAP 1613/5	2.07	2.07	0.09	0.01	4.40	1.86	2.22	21
		CCAP 1613/6	2.09	2.08	0.04	0	2.10	1.98	2.17	21
		CCAP 1613/7	1.49	1.49	0.01	0	1.14	1.46	1.52	21
		CCAP 1613/8	1.64	1.63	0.05	0.01	3.37	1.55	1.73	21
		CCAP 1613/9	1.85	1.84	0.02	0	1.31	1.82	1.90	21
		CCAP 1613/10	1.82	1.83	0.03	0	2.02	1.77	1.88	21
		CCAP 1613/11	1.87	1.86	0.03	0	1.80	1.82	1.95	21
		CCAP 1613/12	1.79	1.80	0.03	0	2.13	1.71	1.88	21
	Coleps hirtus	CCAP 1613/14	1.93	1.94	0.12	0.02	6.40	1.68	2.14	21
	Nolandia nolandi	CCAP 1613/15	2.01	1.97	0.11	0.02	5.90	1.80	2.24	21

Character	Species	Strain number	Number of plates	Number of windows	Anterior main plate Number of windows - (n)	Anterior secondary plate - Number of windows - (n)	Posterior main plate - Number of windows - (n)
General characteristics	*Coleps virdis*	CIL-2017/5	6	14-16	5 (21)	2 (21)	5 (21)
		CIL-2017/6	6	14-16	5 (21)	2 (21)	4 (21)
		CIL-2017/7	6	14-16	5 (18)<br>5-6 (3)	2 (21)	4-5 (5)<br>5 (16)
		CIL-2017/8	6	14-16	5 (21)	2 (21)	4-5 (1)<br>5 (20)
		CIL-2017/9	6	14-16	4 (21)	2 (21)	4 (21)
		CIL-2017/10	6	14-16	4-5 (1)<br>5 (20)	2 (21)	5 (21)
		CIL-2017/13	6	14-16	4-5 (5)<br>5 (15)<br>5-6 (1)	2 (17)<br>2-3 (4)	4 (1)<br>4-5 (8)<br>5 (12)
		CCAP 1613/3	6	12-13	4 (19)<br>4-5 (2)	2 (21)	4 (21)
		CCAP 1613/4	6	12	4 (18)<br>4-5 (3)	2 (21)	4 (21)
		CCAP 1613/5	6	12	4 (21)	2 (21)	4 (21)
		CCAP 1613/6	6	12	4 (21)	2 (21)	4 (21)
		CCAP 1613/7	6	14-16	4-5 (1)<br>5 (18)<br>5-6 (2)	2 (21)	4-5 (4)<br>5 (17)
		CCAP 1613/8	6	12-14	4 (3)<br>4-5 (16)<br>5 (2)	2 (7)<br>2-3 (12)<br>3 (2)	4 (2)<br>4-5 (15)<br>5 (4)
		CCAP 1613/9	6	12	4 (21)	2 (21)	4 (21)
		CCAP 1613/10	6	12-14	3-4 (1)<br>4 (20)	2 (21)	4 (21)
		CCAP 1613/11	6	14-16	4-5 (1)<br>5 (20)	2 (21)	4 (3)<br>4-5 (10)<br>5 (8)
		CCAP 1613/12	6	12-14	4 (21)	2 (21)	4 (21)
	Coleps hirtus	CCAP 1613/14	6	12-13	3-4-5 (1)<br>4 (17)<br>4-5 (2)<br>5 (1)	2 (21)	4 (11)<br>4-5 (6)<br>5 (4)
	Nolandia nolandi	CCAP 1613/15	6	12-14	3-4 (1)<br>4 (2)<br>4-5 (16)<br>5 (1)<br>5-6 (1)	2 (21)	4 (3)<br>4-5 (12)<br>5 (3)<br>6 (2)

Character	Species	Strain number	Posterior secondary plate - Number of windows - (n)	Number of caudal cilia	Number of anterior spines - (n)	Number of posterior spines - (n)	Window type
General characteristics	*Coleps virdis*	CIL-2017/5	2 (21)	1 (21)	0 (7)<br>1 (14)	3 (4)<br>4(17)	*hirtus*-type
		CIL-2017/6	2 (21)	1 (21)	0 (7)<br>1 (14)	3 (5)<br>4(16)	*hirtus*-type
		CIL-2017/7	2 (21)	1 (21)	0 (8)<br>1 (13)	3 (4)<br>4(17)	*hirtus*-type
		CIL-2017/8	2 (21)	1 (21)	1 (21)	2 (2)<br>3 (3)<br>4 (12)<br>5 (1)	*hirtus*-type
		CIL-2017/9	2 (21)	1 (21)	0 (1)<br>1 (20)	3 (6)<br>4(15)	*hirtus*-type
		CIL-2017/10	2 (21)	1 (21)	0 (4)<br>1 (16)<br>2 (1)	3 (2)<br>4(19)	*hirtus*-type
		CIL-2017/13	2 (21)	1 (21)	0 (13)<br>1 (7)<br>2 (1)	0 (1)<br>1 (1)<br>2 (4)<br>3 (6)<br>4 (9)	*hirtus*-type
		CCAP 1613/3	2 (21)	1 (21)	0 (21)	0 (21)	*hirtus*-type
		CCAP 1613/4	2 (21)	1 (21)	0 (21)	0 (20)<br>1 (1)	*hirtus*-type
		CCAP 1613/5	2 (21)	1 (21)	0 (21)	1 (5)<br>2 (6)<br>3 (6)<br>4 (4)	*hirtus*-type
		CCAP 1613/6	2 (21)	1 (21)	1 (21)	2 (1)<br>3 (13)<br>4 (7)	*hirtus*-type
		CCAP 1613/7	2 (20)<br>3 (1)	1 (21)	0 (8)<br>1 (13)	1 (2)<br>2 (5)<br>3 (5)<br>4 (9)	*hirtus*-type
		CCAP 1613/8	2 (20)<br>2-3 (1)	1 (21)	0 (2)<br>1 (18)<br>2 (1)	2 (7)<br>3 (13)<br>5 (1)	*hirtus*-type
		CCAP 1613/9	2 (21)	1 (21)	0 (13)<br>1 (8)	0 (1)<br>1 (2)<br>2 (8)<br>3 (10)	*hirtus*-type
		CCAP 1613/10	2 (21)	1 (21)	0 (20)<br>1 (1)	0 (8)<br>1 (9)<br>2 (4)	*hirtus*-type
		CCAP 1613/11	2 (21)	1 (21)	0 (19)<br>1 (2)	2 (3)<br>3 (9)<br>4 (9)	*hirtus*-type
		CCAP 1613/12	2 (21)	1 (21)	0 (18)<br>1 (3)	1 (2)<br>2 (7)<br>3 (7)<br>4 (5)	*hirtus*-type
	Coleps hirtus	CCAP 1613/14	2 (21)	1 (21)	0 (21)	1 (5)<br>2 (6)<br>3 (6)<br>4 (4)	*hirtus*-type
	Nolandia nolandi	CCAP 1613/15	2 (15)<br>2-3 (1)<br>3 (5)	1 (21)	0 (21)	0 (1)<br>1 (6)<br>2 (10)<br>3 (4)	*nolandi*-type

Character	Species	Strain number	Shape of ridge	Body shape	Presence of green algal endosymbionts - (n)
General characteristics	*Coleps virdis*	CIL-2017/5	Wing-like	Barrel-shaped	Yes (21)
		CIL-2017/6	Wing-like	Barrel-shaped	Yes (18)<br>No (3)
		CIL-2017/7	Wing-like	Barrel-shaped	Yes (21)
		CIL-2017/8	Wing-like	Barrel-shaped	Yes (16)<br>No (5)
		CIL-2017/9	Wing-like	Barrel-shaped	No (21)
		CIL-2017/10	Wing-like	Barrel-shaped	Yes (4)<br>No (17)
		CIL-2017/13	Wing-like	Barrel-shaped	Yes (21)
		CCAP 1613/3	Not wing-like	Barrel-shaped	No (21)
		CCAP 1613/4	Not wing-like	Barrel-shaped	Yes (21)
		CCAP 1613/5	Not wing-like	Barrel-shaped	Yes (11)<br>No (10)
		CCAP 1613/6	Wing-like	Barrel-shaped	No (21)
		CCAP 1613/7	Wing-like	Barrel-shaped	Yes (21)
		CCAP 1613/8	Wing-like	Barrel-shaped	No (21)
		CCAP 1613/9	Wing-like	Barrel-shaped	No (21)
		CCAP 1613/10	Wing-like	Barrel-shaped	No (21)
		CCAP 1613/11	Wing-like	Pyriform	Yes (21)
		CCAP 1613/12	Wing-like	Barrel-shaped	No (21)
	Coleps hirtus	CCAP 1613/14	Not wing-like	Barrel-shaped	No (21)
	Nolandia nolandi	CCAP 1613/15	Wing-like	Cylindrical	No (21)

Table S3: List of GenBank entries found with the BLASTn search algorithm (100% coverage, >97% identity) using the V4 region (SSU) and the respective haplotype. Accession numbers marked in green are the strains investigated in this study.

Accession #	Haplotype	Origin	Habitat	Reference
**MT253670**	1a	UK	freshwater	this study
**MT253671**	1a	UK	freshwater	this study
**MT253672**	1a	UK	freshwater	this study
**MT253673**	1a	Switzerland	freshwater	this study
**MT253674**	1a	Austria	freshwater	this study
**MT253675**	1a	Austria	freshwater	this study
**MT253676**	1a	Austria	freshwater	this study
**MT253677**	1a	Austria	freshwater	this study
**MT253678**	1a	Austria	freshwater	this study
**MT253679**	1a	Austria	freshwater	this study
**MT253680**	1a	Austria	freshwater	this study
**MT253681**	1a	Austria	freshwater	this study
**MT253682**	1a	Switzerland	freshwater	this study
**MT253683**	1a	Austria	freshwater	this study
**MT253684**	1a	Germany	freshwater	this study
**MT253686**	1a	Switzerland	freshwater	this study
**AM292312**	1a	Germany	freshwater	Barth et al. (2008)
**LS999899**	1a	Switzerland	freshwater	Pitsch et al. (2019)
**JN090894**	1a	Greece	freshwater	Oikonomou et al. (2012)
**EU446361**	1a	Mediterranean Sea	marine	Alexander et al. (2009)
**EU024974**	1a	Austria	freshwater	Auinger et al. (2008)
**FJ765380**	1a	South Korea	freshwater	Joo et al. (2010)
**LN870142**	1b	Italy	freshwater	Rossi et al. (2016)
**LN870060**	1b	Italy	freshwater	Rossi et al. (2016)
**LN869945**	1b	Italy	freshwater	Rossi et al. (2016)
**LN870149**	1b	Italy	freshwater	Rossi et al. (2016)
**LN870054**	1b	Italy	freshwater	Rossi et al. (2016)
**EU446396**	1c	Mediterranean Sea	marine	Alexander et al. (2009)
**U97109**	1d	unknown	unknown	unpublished
**MT253685**	1e	Austria	freshwater	this study
**MT253687**	2a	Austria	freshwater	this study
**LN870048**	2a	Italy	freshwater	Rossi et al. (2016)
**LN870110**	2a	Italy	freshwater	Rossi et al. (2016)
**LN870063**	2a	Italy	freshwater	Rossi et al. (2016)
**LN870036**	2a	Italy	freshwater	Rossi et al. (2016)
**LN870025**	2a	Italy	freshwater	Rossi et al. (2016)
**LN870011**	2a	Italy	freshwater	Rossi et al. (2016)
**AM292311**	2a	Germany	freshwater	Barth et al. (2008)
**DQ487194**	2a	USA	freshwater	Duff et al. (2008)
**HM140399**	2b	South Korea	freshwater	unpublished
**LN870155**	2c	Italy	freshwater	Rossi et al. (2016)
**KF639909**	2c	USA	freshwater	Dunthorn et al. (2014)
**X76646**	2d	USA	freshwater	Stechmann et al. (1998)

References:

Alexander, E., Stock, A., Breiner, H.-W., Behnke, A., Bunge, J., Yakimov, M. M. & Stoeck, T. Microbial eukaryotes in the hypersaline anoxic L'Atalante deep-sea basin. *Environ. Microbiol.***11**, 360-381 (2009).

Auinger, B. M., Pfandl, K., Boenigk, J. Improved methodology for identification of protists and microalgae from plankton samples preserved in Lugol's iodine solution: combining microscopic analysis with single-cell PCR. *Appl. Environ. Microbiol.* **74**, 2505-2510 (2008).

Barth, D., Tischer, K., Berger, H., Schlegel, M. & Berendonk, T. High mitochondrial haplotype diversity of *Coleps* sp. (Ciliophora: Prostomatida). *Environ. Microbiol.* **10**, 626-634 (2008).

Duff, R.J., Ball, H., Lavrentyev, P. J. Application of combined morphological-molecular approaches to the identification of planktonic protists from environmental samples. *J. Eukaryot. Microbiol.* **55**, 306-312 (2008).

Dunthorn, M., Hall, M., Foissner, W., Stoeck, T., Katz, L. A. Broad taxon sampling of ciliates using mitochondrial small subunit ribosomal DNA. *Acta Protozool.* **53**, 207-213 (2014).

Joo, S., Lee, S.R., Park, S. Monitoring of phytoplankton community structure using terminal restriction fragment length polymorphism (T-RFLP). *J. Microbiol. Methods* **81**, 61-68 (2010).

Oikonomou, A., Katsiapi, M., Karayanni, H., Moustaka-Gouni, M., Kormas, K. A. Plankton microorganisms coinciding with two consecutive mass fish kills in a newly reconstructed lake. *Sci. World J*. **2012**, 504135 (2012).

Pitsch, G., Bruni, E. P., Forster, D., Qu, Z., Sonntag, B., Stoeck T. & Posch, T. Seasonality of planktonic freshwater ciliates: are analyses based on V9 regions of the 18S rRNA gene correlated with morphospecies counts? *Front. Microbiol.* **10**, 248 (2019).

Rossi, A., Boscaro, V., Carducci, D., Serra, V., Modeo, L., Verni, F., Fokin, S. I., Petroni, G. Ciliate communities and hidden biodiversity in freshwater biotopes of the Pistoia province (Tuscany, Italy). *Eur. J. Protist.* **53**, 11-19 (2016).

Stechmann, A., Schlegel, M., Lynn, D. H. Phylogenetic relationships between prostome and colpodean ciliates tested by small subunit rRNA sequences. *Mol. Phylogenet. Evol.* **9**, 48-54 (1998).

Table S4: Geography and hydrological parameters of both lakes (Lake Mondsee, Austria and Lake Zurich, Switzerland).

Lake	Mondsee (LM)	Zurich (LZ)
Country	Austria	Switzerland
Coordinates sampling site	47°50′N, 13°23′E	47°19.3′N, 8°33.9′E
Elevation (m a.s.l.)	481	406
Surface area (km²)	14.2	66.2
Average depth (m)	36	50
Maximum depth (m)	68	136
